# An Overview of Circulating Pulmonary Arterial Hypertension Biomarkers

**DOI:** 10.3389/fcvm.2022.924873

**Published:** 2022-07-14

**Authors:** Joana Santos-Gomes, Inês Gandra, Rui Adão, Frédéric Perros, Carmen Brás-Silva

**Affiliations:** ^1^UnIC@RISE, Department of Surgery and Physiology, Faculty of Medicine of the University of Porto, Porto, Portugal; ^2^Paris-Porto Pulmonary Hypertension Collaborative Laboratory (3PH), UMR_S 999, INSERM, Université Paris-Saclay, Paris, France; ^3^Université Paris–Saclay, AP-HP, INSERM UMR_S 999, Service de Pneumologie et Soins Intensifs Respiratoires, Hôpital de Bicêtre, Le Kremlin Bicêtre, France; ^4^Faculty of Nutrition and Food Sciences, University of Porto, Porto, Portugal

**Keywords:** biomarkers, pulmonary arterial hypertension, pulmonary hypertension, prognosis, diagnosis, circulating levels

## Abstract

Pulmonary arterial hypertension (PAH), also known as Group 1 Pulmonary Hypertension (PH), is a PH subset characterized by pulmonary vascular remodeling and pulmonary arterial obstruction. PAH has an estimated incidence of 15–50 people per million in the United States and Europe, and is associated with high mortality and morbidity, with patients' survival time after diagnosis being only 2.8 years. According to current guidelines, right heart catheterization is the gold standard for diagnostic and prognostic evaluation of PAH patients. However, this technique is highly invasive, so it is not used in routine clinical practice or patient follow-up. Thereby, it is essential to find new non-invasive strategies for evaluating disease progression. Biomarkers can be an effective solution for determining PAH patient prognosis and response to therapy, and aiding in diagnostic efforts, so long as their detection is non-invasive, easy, and objective. This review aims to clarify and describe some of the potential new candidates as circulating biomarkers of PAH.

## Introduction

Pulmonary hypertension (PH) is classified into five groups based on etiology and pathogenesis (1). Patients in the first group are considered to have pulmonary arterial hypertension (PAH) (2, 3), a chronic and severe cardiopulmonary disease with a poor prognosis (4). According to the latest revised World Symposium on Pulmonary Hypertension, PAH can be subclassified into 7 distinct groups, as described in Table 1 (5). Although PAH is considered a rare disease, it is estimated to have an incidence of 15–50 people per million in the United States and Europe. Most PAH cases (52.6%) are idiopathic, heritable, and/or anorectic-induced PAH (6). The incidence of PAH can, however, be associated with other morbidities (7).

**Table 1:** Updated clinical classification of pulmonary hypertension.

**1. Pulmonary arterial hypertension**	
	1.1 Idiopathic PAH
	1.2 Heritable PAH
	1.3 Drug- and toxin-induced PAH
	1.4 PAH associated with other conditions
	1.4.1 Connective tissue disease
	1.4.2 HIV infection
	1.4.3 Portal hypertension
	1.4.4 Congenital heart disease
	1.4.5 Schistosomiasis
	1.5 PAH long-term responders to calcium channel blockers
	1.6 PAH with overt features of venous/capillaries (PVOD/PCH) involvement
	1.7 Persistent PH of the newborn syndrome
**2. PH due to left heart disease**	
	2.1 PH due to heart failure with preserved LVEF
	2.2 PH due to heart failure with reduced LVEF
	2.3 Valvular heart disease
	2.4 Congenital/acquired cardiovascular conditions leading to post-capillary PH
**3. PH due to lung diseases and/or hypoxia**	
	3.1 Obstructive lung disease
	3.2 Restrictive lung disease
	3.3 Other lung disease with mixed restrictive/obstructive pattern
	3.4 Hypoxia without lung disease
	3.5 Developmental lung disorders
**4. PH due to pulmonary artery obstructions**	
	4.1 Chronic thromboembolic PH
	4.2 Other pulmonary artery obstructions
**5. PH with unclear and/or multifactorial mechanisms**	
	5.1 Hematological disorders
	5.2 Systemic and metabolic disorders
	5.3 Others
	5.4 Complex congenital heart disease

*PH, pulmonary hypertension; PAH, pulmonary arterial hypertension; HIV, Human immunodeficiency virus; PVOD, pulmonary veno-occlusive disease; PCH, pulmonary capillary hemangiomatosis; LVEF, left ventricle ejection fraction*.

PAH is characterized by excessive pulmonary vascular remodeling, which involves medial hypertrophy—an early event in PAH and even reversible, appearing in all PAH subgroups -, proliferative and fibrotic changes of the intima, adventitious thickening, and thrombosis *in situ* (1–3, 8–11). The mechanisms behind these transformations are not fully understood (12)—several pathophysiological processes are entailed, such as migration and proliferation of pulmonary arterial smooth muscle cells (PASMCs) and endothelial cells (ECs) (1, 2), endothelial dysfunction, endothelial-to-mesenchymal transition (13), enhanced adventitial pulmonary artery fibroblast proliferation, migration, and differentiation (14), inflammation (15, 16) and oxidative stress (2, 3, 8, 9). Particularly, ECs may be involved in synthesizing growth factors that stimulate non-cellular matrix deposition and smooth muscle hypertrophy, contributing to the formation of plexiform lesions (1, 4, 10, 17). Moreover, necrotic and fibrotic tissue, as well as inflammatory cells, can also accumulate in the arterial wall, resulting in arteritis (1, 2, 4). Finally, there are changes in the production of vasoactive molecules, namely nitric oxide (NO), prostacyclins and endothelin-1 (ET-1) (2, 18), contributing to endothelial dysfunction and vasoconstriction.

All these alterations culminate in pulmonary arterial obstruction, with an increase in pulmonary vascular resistance (PVR), which will lead to right ventricular overload and, eventually, right ventricle (RV) failure (1, 9, 19)—the major cause of PAH mortality and morbidity (8). Early studies, before the introduction of PAH-specific therapies, suggested that patients' survival after diagnosis was only 2.8 years (20, 21).

Patients present with symptoms which reflect RV dysfunction and are non-specific (2, 3, 18). Because of this, the diagnosis is often delayed for ≥2 years, with many patients being diagnosed only in advanced stages (22). To characterize PAH, current guidelines require patients to undergo a right heart catheterization, with measurements of mean pulmonary artery pressure (mPAP) > 20 mmHg, pulmonary artery wedge pressure ≤ 15 mmHg and a PVR ≥ 3 Wood Units at rest, in the absence of other causes (5).

Upon establishing a diagnosis of PAH, adequate treatment must be started. Therapy for PAH patients has evolved considerably in the past decades, in parallel with improvements in patient survival and quality of life (21). A multidimensional approach is recommended, involving general measures (supervised physical rehabilitation, infection prevention and psychosocial support), supportive therapy (such as diuretics and supplementary oxygen) and PAH targeted drug therapy (17). Specific PAH therapies are targeted at the three main molecular pathways altered in dysfunctional pulmonary endothelium. Endothelin receptor antagonists (ERAs) modulate the endothelin (ET) pathway; phosphodiesterase type 5 (PDE-5) inhibitors and soluble guanylate cyclase stimulators act *via* the NO pathway; and prostacyclin analogs and prostacyclin receptor agonists act in the prostanoid pathway. These agents act mainly by inducing pulmonary vasodilation. Initial treatment usually entails dual combination therapy, and patients are regularly evaluated for disease control and adjustment of treatment accordingly (17).

Right heart catheterization is the current gold standard for establishing both disease diagnosis and prognosis, particularly when considering the adjustment of treatment. However, its invasiveness prevents it from being used in routine clinical practice or patient follow-up, being replaced by measurements of systolic pulmonary artery pressure (PAP) and transthoracic echocardiography (23).

Thus, it is essential to find non-invasive techniques for disease monitoring. Biomarkers can be an effective solution for establishing diagnosis and prognosis, and evaluating response to therapy, so long as their detection is non-invasive, easy, and objective. This review aims to clarify and describe some of the potential candidates as a biomarker for the diagnosis and prognosis of PAH.

## What is a Biomarker?

By definition, a biomarker is “a characteristic that is measured objectively and evaluated as an indicator of normal biological processes, pathogenic processes or pharmacological responses to a therapeutic intervention” (12, 24). More succinctly, we can define a biomarker as a molecular change in tissues and/or body fluids as result of a disease process (12). Ideally, a biomarker should represent clinical outcomes, that is, reflect how the patient feels and what stage of disease he is in; it should also serve as tool for diagnosis and prognosis, and as a therapeutic marker, providing information on the patient's response to a specific treatment (12, 25). Moreover, it must display a series of properties that make it ideal, namely, high sensitivity and specificity, easiness to obtain/collect and measure, total availability, non-invasive, and it must also be a sign of disease activity (risk stratification, responsiveness to treatment, anticipation of clinical worsening) or a treatment target (12, 25). The search for ideal biomarkers is constant and, in situations where an ideal candidate is not yet known, the one(s) considered to be more accessible, cheaper, and easier to measure is (are) used. However, as these can be less sensitive and less specific, they should not be used alone as a clinical decision tool, and a set of factors must be used to make a decision (25).

**Figure 1:**
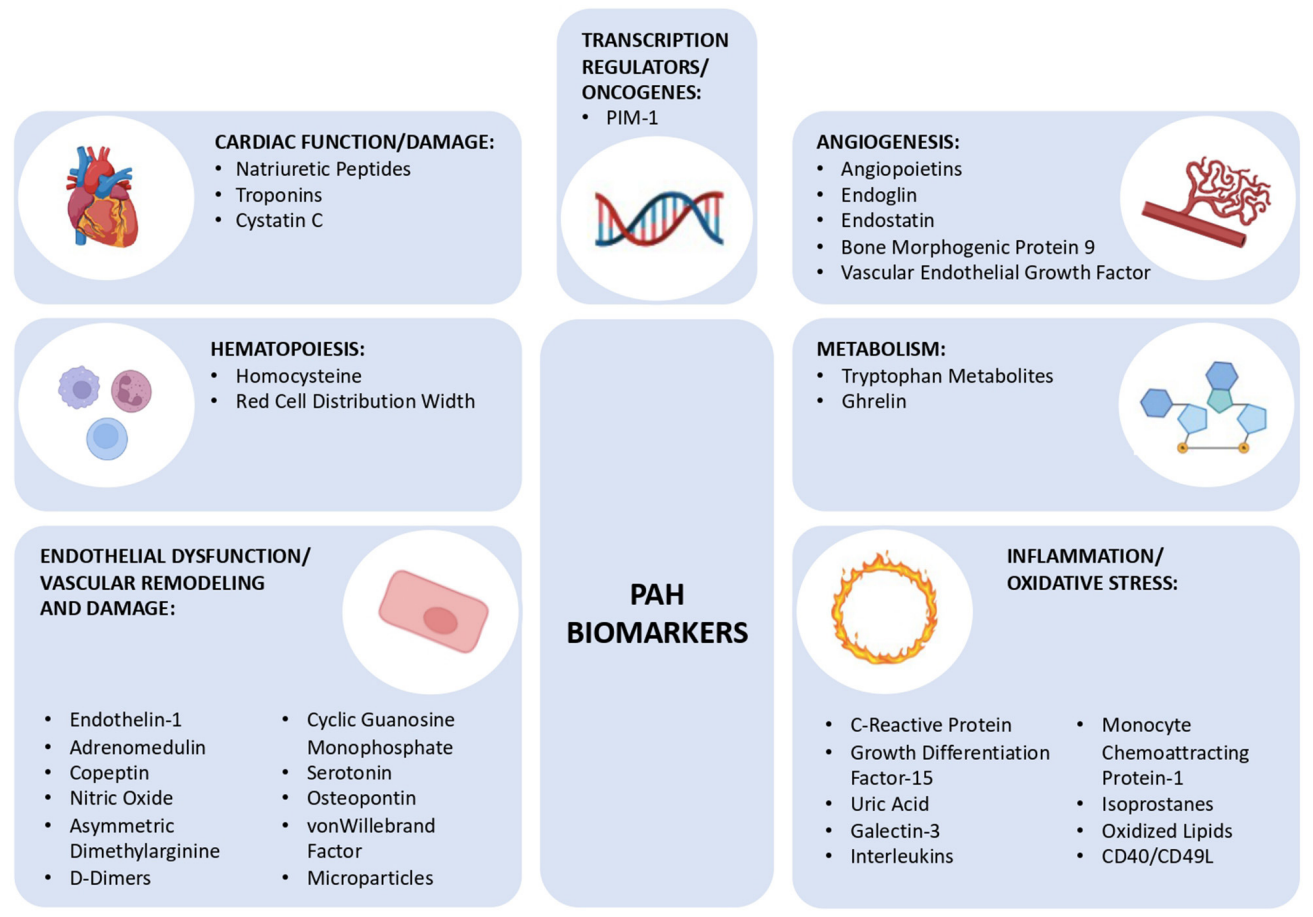
Schematic diagram showing the different group of biomarkers for pulmonary arterial hypertension discussed in this review, subclassified into 7 groups: cardiac function/damage, hematopoiesis, endothelial dysfunction/vascular remodeling and damage, angiogenesis, metabolism, inflammation/oxidative stress, and transcription regulators/oncogenes. PIM-1, Moloney Murine Leukemia Provirus Integration Site.

Although a PAH biomarker that is detected by a single and simple test is not yet known, there are already several well-known and well-defined biomarkers that could prove to be potent diagnostic and prognostic indicators in the future (23, 26). These biomarkers can be categorized according to the (patho)physiological mechanism they are associated with, which reflects the complexity of this syndrome: endothelial function, inflammation, oxidative stress, cardiac function, myocardial injury, metabolism, and gene expression (Figure 1).

## Biomarkers of Cardiac Function/Damage

### Natriuretic Peptides

Natriuretic peptides are a family of genetically distinct hormones that share a similar molecular structure (12). This family includes atrial natriuretic peptide (ANP), brain natriuretic peptide (BNP), C-type natriuretic peptide (3, 27) and dendroaspis natriuretic peptide, a D-type natriuretic peptide, each having specific functions (27). They are fundamental in cardiac homeostasis (28), as they are involved in the regulation of blood volume and blood pressure through their diuretic, natriuretic, vasodilatory (3, 29) and kaliuretic (27) activities, in addition to inhibiting the renin-angiotensin-aldosterone system (27, 29) and regulating the proliferation of ECs (27). These hormones are secreted essentially by the heart, kidneys, and brain (3).

ANP and BNP represent the main hormones of the natriuretic peptide system (12, 30), both are released from cardiac myocytes, ANP is released essentially from atrial tissue, while BNP is released from ventricular tissue. Both are secreted in response to increased heart pressure and volume overload (31). BNP, being secreted by the ventricular tissue, is more sensitive to ventricular diseases when compared to ANP (12).

BNP is a transcription product of the *NPPB gene*, first forming a precursor of 134 amino acids (aa), preproBNP, which is enzymatically cleaved, forming proBNP (3, 30). ProBNP is cleaved into two distinct fragments: an active peptide, mature 32 aa BNP and an inactive N-terminal fragment, the N-terminal prohormone (NT-proBNP) (3, 27, 30). BNP acts by binding to receptor A, which is mainly expressed in the kidneys, adrenal, lung, terminal ileum, aorta, and adipose tissue (3). The activation of this receptor induces an increase in the levels of cyclic guanosine monophosphate (cGMP), which triggers a vasodilator, natriuretic response and inhibits aldosterone (3). On the other hand, although the function of NT-proBNP is not yet clear (3), it is eliminated by the kidneys and, therefore, its plasma levels increase significantly when there are changes in renal function; the same does not happen with BNP (30). Mature BNP has a short plasma half-life (about 22 min), while NT-proBNP has a 2-h half-life (30) and therefore has greater stability (3, 12) and it becomes easier to measure (12). The levels of cardiac and circulating BNP increase significantly in response to hypertrophy and/or ventricular overload, demonstrating that BNP is an excellent marker of ventricular dysfunction (28).

In PH pathophysiology: BNP is elevated in several forms of PAH, including idiopathic PAH (IPAH) (32), PAH associated with connective tissue diseases (CTD-PAH) (33), congenital systemic-pulmonary shunts (34), PH associated with lung diseases (35), PH with chronic obstructive pulmonary disease (COPD) (28), chronic thromboembolic PH (CTEPH) (29) and PH associated with acute pulmonary embolism (36). BNP levels, in several studies, are closely correlated with New York Heart Association (NYHA) functional class, 6-min walk distance (6MWD) test, and hemodynamic parameters (3, 29, 32, 37). Nagaya et al. it also demonstrated that BNP levels correlate positively with mPAP and inversely with cardiac output, showing a strong correlation with total pulmonary resistance (38). NT-proBNP is also increased in different forms of PAH such as IPAH (28) and systemic sclerosis-associated PAH (SSc-PAH) (39). In the latter case, NT-proBNP correlates with mPAP, PVR, mean right atrial pressure (mRAP) and cardiac index (3), and it can effectively work as a predictor of survival in PAH (23). ANP levels are also increased in IPAH (40) and CTEPH. Like BNP, ANP levels are positively correlated with the mPAP and inversely with cardiac output (38). However, ANP has a short half-life in humans subjects—about 2 min (41, 42)—while BNP has a much longer half-life (43), making BNP a better candidate as biomarker (3).

Thus, BNP and NT-proBNP are sensitive and specific biomarkers for risk stratification in PAH (they are, so far, the only biomarker included in current guidelines) (23). They are also sensitive markers of RV dysfunction and of treatment efficacy (23, 26, 28). In addition, BNP, as a pulmonary vasodilator and antihypertrophic agent, has a therapeutic potential to alleviate pulmonary vascular remodeling (28). However, there are always factors that must be considered, namely the patients' gender and age, the presence of left heart disease, renal dysfunction, and obesity (12, 26). In any situation of left heart disease, BNP levels should not be used as predictors of diagnosis or prognosis for PAH (30).

### Troponin

Cardiac troponins (cTn) are a set of 3 proteins—troponin C, troponin I (cTnI) and troponin T (cTnT) -, whose main function is to regulate the thin actin filaments of the heart muscle. The level of troponins is closely related to damage to the myocardium since the rupture of the cardiomyocyte membranes causes its release into the peripheral blood. Therefore, it is possible to detect it by highly sensitive assays in plasma (12, 26). The measurement of cTnT and cTnI is essential in the diagnosis and prognosis of patients with acute coronary syndrome, as well as of all pathologies associated with myocardial lesions which present increased levels of cardiac troponins, as is the case of myocarditis (12).

In PH pathophysiology: several studies linked cTnT to the poor prognosis of PH. Torbicki et al. demonstrated that in patients with PAH and CTEPH, cTnT levels are increased, which is probably explained by the damage to the RV myocardium. However, in this study, only 14% of patients (8 out of 56 patients) had an elevation of cTnT levels—since the test used to measure cTnT levels only allows detection of levels >0.01 ng /ml. Nonetheless, when comparing cTnT(+) *vs*. cTnT(-) patients, it was found that they had similar pulmonary hemodynamics, but cTnT(+) patients had a higher heart rate, lower mixed venous saturation of oxygen (SvO_2_), higher serum NT-proBNP and less resistance to exercise (measured by 6MWD) (44). In addition, in patients undergoing treatment for PH, it was found that cTnT levels decrease, becoming even undetectable; in contrast, levels increase with the progression of the disease (44).

More recent studies present tests with greater sensitivity to cTn, with significantly lower detection levels. Heresi et al. demonstrated, using an immunoanalyzer with a detection limit of <0.008 ng/mL, that cTnI elevation was detected in 25% of patients with PAH. cTnI(+) patients, when compared to cTnI(-) patients, had a higher functional class, larger right atrial area, lower 6MWD, and higher levels of BNP and C-reactive protein (CRP). Furthermore, the survival of cTnI(+) patients was significantly lower than that of cTnI(-) patients (44% *vs*. 85%) (45). Filusch et al. evaluated cTnT levels in patients with PAH, comparing the conventional assay with the high-sensitive cTnT (hsTnT) assay, with a detection limit lower than 2 pg/ml. In 90.9% of patients, cTnT was detectable using the hsTnT assay *vs*. 30.9% using the conventional assay. In addition, the hsTnT assay measurements were significantly associated to systolic RV dysfunction and an impaired 6MWD. Furthermore, hsTnT predicted a World Health Organization (WHO) functional class of II or higher better than NT-proBNP, and predicted death as effectively as NT-proBNP (46). In another study, using the new hsTnI assay, cTnI levels were detectable in 95% of patients with PH, including PAH patients. Higher cTnI levels are associated with higher BNP levels, lower 6MWD, more severe hemodynamic abnormalities, and cardiac magnetic resonance imaging abnormalities (47).

Although cTn are also related to some markers on the left side of the heart (47), which can be a confounding factor, studies indicated that they can be used as indicators of disease severity. Furthermore, use of the new hsTn assays provides new prognostic information and these assays have the potential to detect more patients, at higher risk, and therefore facilitate risk stratification. Nevertheless, there are confounding factors that must be considered, namely, the presence of concomitant left heart disease or renal failure (12, 26).

### Cystatin C

Cystatin C (CysC) is a 13 kDa non-glycosylated protein (48, 49), is produced at a constant rate by all investigated nucleated cells (50), and it is a member of the cystatin superfamily that comprises inhibitors of cysteine proteinases (51). In recent years, the importance of CysC has increased due to its free filtration in the glomerulus, complete reabsorption and catabolism in the proximal tubule and lack of tubular secretion. It is believed that the plasma concentration of CysC depends almost completely on the glomerular filtration rate, thus making it an ideal marker of renal function (48, 49). In addition, it is more sensitive than serum creatine since it detects smaller reductions in glomerular filtration rate (48, 49). Several studies have shown that CysC can also function as a cardiovascular risk marker since it predicts left heart failure (HF) and cardiovascular mortality in general (48, 49).

In PH pathophysiology: Fenster et al. showed that patients with PAH have abnormally high serum CysC levels and that these levels correlate with RV function (52). These studies show that RV systolic pressure is highly elevated in PAH patients and that it correlates positively with CysC serum levels. Furthermore, RV end-diastolic volume, RV end-systolic volume, mass index, strain and strain rate are positively correlated with CysC levels, and RV ejection fraction is negatively correlated (52). Thus, CysC can be a sensitive biomarker for assessing PAH and it also has additional advantages over standard biomarkers, BNP and NT-proBNT, of being independent of muscle mass, age, and gender (50).

## Biomarkers of Hematopoiesis

### Homocysteine

Homocysteine is a sulfur-containing intermediate product of the normal metabolism of methionine, an essential amino acid, obtained essentially from animal protein (53). Homocysteine must be recycled by a route that requires folic acid and vitamins B_6_ and B_12_. Alterations in this pathway, such as deficits in vitamin B_6_ and B_12_ levels, genetic defects, or polymorphisms in the main enzymes of this pathway, can promote an increase in homocysteine levels, which can be harmful (53). Homocysteine is an inhibitor of dimethylarginine dimethylaminohydrolase, an enzyme that metabolizes asymmetric dimethylarginine (ADMA); ADMA is an endogenous inhibitor of the NO synthase (NOS) pathway. Thus, an increase in homocysteine levels, known as hyperhomocysteinemia, results in a decrease in NO bioavailability. It is believed that this decrease in NO happens because homocysteine inhibits the activity of dimethylarginine dimethylaminohydrolase, promoting the accumulation of ADMA and, consequently, a decrease in the production of NO by ECs (54). Furthermore, it is also believed that homocysteine is involved in the oxidative degradation of NO (54, 55). Since homocysteine essentially affects ECs, coagulation, and platelet function, it is possible to understand why the endothelial vasodilator function is impaired in individuals with hyperhomocysteinemia, as well as associated with platelet dysfunction, facilitating coagulation; thus, increasing the risk of cardiovascular diseases and an acceleration in vascular diseases (54, 55).

In PH pathophysiology: studies show that total plasma homocysteine levels—includes homocysteine and its oxidized derivatives—mixed homocysteine-cysteine disulphide and protein-bound homocysteine—are higher in patients with PAH compared to healthy controls (53). Furthermore, Sanli et al. showed that homocysteine levels are higher in patients with PAH associated with congenital heart disease (CHD-PAH), as are ADMA levels, but they did not correlate with hemodynamic factors (56). Thus, both studies suggest that homocysteine may be an important factor in the development of PAH, which can be well-justified by the endothelial damage caused by hyperhomocysteinemia. However, more studies should be done, since the sample size in both existing studies is relatively low (56).

### Red Cell Distribution Width

Red blood cell distribution width (RDW) is a parameter that reflects the variation in the size of circulating red blood cells; it is routinely measured in a complete blood count, and it is essential in the differential diagnosis of anemia (57–59). Several studies showed that RDW values can be useful in predicting malignant tumors (60, 61). RDW is associated with several pathophysiological mechanisms, such as inflammation, iron metabolism, renal dysfunction, nutritional status, and oxidative stress which result in a decrease of erythropoietic output (62). Increased levels of RDW are closely associated with impaired erythropoiesis or erythrocyte degradation (63). RDW has proven to be a promising predictor of the clinical outcome of renal diseases, cardiovascular diseases, pulmonary diseases (57–59), such as HF, PH of various etiologies (58), acute myocardial infarction, community-acquired pneumonia, pulmonary embolism, and COPD; it has also been shown as a predictor of mortality in patients with COPD and PAH (64).

In PH pathophysiology: Ulrich et al. demonstrated that RDW is a factor that correlates with survival in PAH; and that iron deficiency is usually seen in patients with PAH (65, 66). RDW levels increase with decreasing iron levels, since the available body iron levels do not respond to the demand for iron from red blood cell synthesis, resulting in varied size of red blood cells (67). Rhodes et al. showed that RDW levels correlate with WHO functional class and 6MWD (68); they also showed that RDW can function as an independent predictor of survival, even when measured in combination with 6MWD, NT-proBNP and other clinical indices (68).

In conclusion, RDW is closely related to the severity of the disease and can be used to predict the survival of patients with IPAH (67, 68). Thus, the use of RDW as a PAH biomarker should be considered for new approaches with multiple biomarkers for PAH stratification; since the levels of RDW in combination with the levels of NT-proBNP showed better detection of high-risk cases, than just using NT-proBNP (68).

## Biomarkers of Endothelial Dysfunction or/and Vascular Remodeling and Damage

### Endothelin-1

The ET system consists of three ET isopeptides (ET-1, ET-2, and ET-3), activating peptidases with different isoforms and two G protein-coupled receptors—ET type A receptor (ETA) and ET type B receptor (ETB) (69). ETs are isopeptides of 21 aa, presenting a high homology and similarity to each other. They are expressed essentially in ECs; however, they are also expressed in cardiac myocytes, pulmonary epithelium, glomerular renal cells, mesangial cells, smooth muscle cells (SMCs), leukocytes, macrophages (7, 70) and fibroblasts (12). ETs are recognized as being the most potent endogenous vasoconstrictors (69) but in addition, they are multifunctional peptides with cytokine or hormone-like activity (71).

ET-1 is the most expressed form in the cardiovascular system (7) and in the pulmonary vasculature (12); it binds to both receptors. ETA and ETB are distributed in various tissues and cells, but their expression is variable; ETA is primarily expressed on vascular smooth muscle cells (VSMCs) (71) and myocytes (7), while ETB is primarily located on ECs (ETB1 receptors), and on VSMCs (ETB2 receptors) (7). Activation of ETA primarily mediates vasoconstriction as well as cell proliferation (69). In contrast, ETB activation in ECs promotes an indirect vasodilator action, anti-proliferation of myocardial and vascular tissues, and renal blood pressure regulation. On the other hand, ETB2 receptors have some vasoconstrictor role, however, their most important action is the ET-1 clearance (69). Vasoconstriction is mediated by activation of phospholipase C, increase in inositol triphosphate and diacylglycerol, with a subsequent increase in intracellular calcium, promoting cell contraction. On the other hand, vasodilation mediated by the activation of endothelial receptors ETB1 stimulates the release of NO and prostacyclins, inducing relaxation of the vascular wall (7). ET-1 clearance occurs by internalization of the receptor to which ET-1 has bound; the lungs clear about 50% of circulating ET-1 (7).

ET-1 induces intense and prolonged vasoconstriction of the pulmonary arteries and veins, even when present in low concentration, and it is also capable of stimulating the proliferation of pulmonary fibroblasts. At the cardiac level, ET-1 is involved in increasing myocardial contractility and heart rate (positive inotropic and chronotropic effect, respectively), in addition to stimulating the production of cytokines, growth factors, and matrix proteins in other tissues (7).

In PH pathophysiology: most patients have increased ET-1 levels (72); and it has been implicated as a mediator of increased vascular tone and vascular remodeling (73). In PAH, there is an evident increase in the expression of ET-1 in the pulmonary vasculature (73) including in the plexiform lesions (7), characteristic of the disease. Also, ET-1 plasma levels are increased and are closely correlated with RAP and pulmonary artery oxygen saturation (5, 74), PVR, and 6MWD (75). Endothelial damage, characteristic of PAH, potentiates the constrictive action of ET-1, causing dysregulation in the ET system (7), and reduces the endothelium's capacity to release vasodilators (72). Thus, this dysregulation and overexpression of ET-1 promote an increase in PVR (7) in part due to the lack of vasodilators (72), and to an abnormal pulmonary vascular remodeling (19). High levels of ET-1 are also associated with an inflammatory response and increased fibrosis (1). The increase in ET-1 plasma levels in PAH may result from an increase in ET-1 release or a reduction in ET-1 clearance by the pulmonary vasculature or even a combination of both factors (1, 72).

Furthermore, inhibition of ET receptors by the action of ERAs is effective in the treatment of PAH, reducing PAP and inhibiting vascular remodeling. Bosentan demonstrated improvements in hemodynamic parameters, in 6MWD, and in WHO functional class in patients with PAH (76); Sitaxsentan showed improvements in 6MWD, WHO functional class, PVR, and cardiac index (77). Ambrisentan improves exercise capacity, WHO functional class, hemodynamics parameters and death in PAH patients; also, it is associated with a low risk of aminotransferase abnormalities (78, 79). In addition, the AMBITION trial showed that dual combination therapy with Ambrisentan and Tadalafil, a PDE-5 inhibitor, reduced the risk of PAH-related hospitalization by 63% compared with just monotherapy (80). Macitentan improves mPAP, RAP, PVR, cardiac index, and the levels of NT-proBNP (81).

Therefore, the ET-1 system plays a fundamental role in the pathology of PAH and can even be used as a prognostic marker of the disease. However, the use of ET-1 as a marker has some limitations that must be considered, since it essentially spreads through vascular structures, its plasma levels do not accurately represent the concentration of ET-1 in the tissue (12, 72). Moreover, demographic characteristics such as ethnicity, sex, and age should always be considered since plasma ET-1 levels are higher in African ethnicity, males, and older age, thus representing potential confounding factors (82).

### Adrenomedullin

Adrenomedullin (ADM) is 52 aa peptide hormone associated with long-lasting pulmonary vasodilator effect (83, 84). ADM was first isolated in 1993 from human phaeochromocytoma (83, 84) and was subsequently found as a circulating hormone (12). ADM is produced by ECs and VSMCs and diffuses between blood and interstitium (83, 85). Despite having receptors and binding sites throughout the body, the density of receptors is higher in the cardiovascular and pulmonary tissues, thus its functioning essentially in these two systems (83, 85). The ADM, in addition to its vasodilating effect, has diuretic and natriuretic effects, inhibits the renin-angiotensin-aldosterone system, and it is involved in angiogenesis and regulation of inflammation (12). Given its vasodilating action, and natriuretic and diuretic effect, it is understandable that ADM is involved in the regulation of body fluid and, therefore, in cardiac homeostasis (85). Also, it has the capability to act as a regulator of pulmonary vascular tone and vascular remodeling (83). Previous studies demonstrated that plasma levels of ADM are increased in patients with hypertension and HF.

In PH pathophysiology: plasma levels of ADM are increased in patients with PAH and increase in proportion to the severity of PH (83, 85). Moreover, ADM levels correlate with clinical parameters including mRAP, 6MWD, and NT-proBNP levels, with ESC/ERS and REVEAL risk scores, and may reflect overall patient survival (86). Studies also showed the importance of ADM as a therapeutic target for PAH—-administration of exogenous ADM resulted in significant hemodynamic improvements (increase in cardiac index and a decrease in PVR) in patients with PAH (83); therefore, acting as a disease-regulating hormone in PAH, in addition to functioning as an alternative prognosis and severity marker (86).

### Copeptin

Copeptin is a 39 aa glycopeptide and it is especially known as arginine vasopressin (AVP)-associated glycopeptide (87). Copeptin, together with AVP and neurophysin II, is derived from a precursor, the pre-pro-vasopressin (87, 88). These three peptides are stoichiometrically secreted by the pituitary gland, and it is possible to use copeptin levels as reporters of AVP levels (87, 88). AVP is produced in the hypothalamus and secreted by the pituitary in response to hemodynamic and/or osmotic stimuli (87). AVP binds to two types of receptors: vasopressin1, which mediate arteriolar vasoconstriction (87, 88), and vasopressin2, which has antidiuretic effects (87), promoting water reabsorption through of the induction of aquaporins in the kidney collecting ducts (88). However, AVP has a short plasma half-life (89) and, since it is unstable, the circulating AVP is mostly linked to platelets (88) and therefore it is impossible to measure. Copeptin is a prognostic marker for several cardiovascular pathologies (12, 87, 88).

In PH pathophysiology: levels of circulating copeptin are increased in PAH patients and are positively correlate with NYHA class and negatively with 6MWD (88). In addition, it was found that the increase in plasma volume and low plasma sodium concentrations are closely related to mortality in patients with PAH (88). Thus, copeptin can function as a predictor of death, transplantation, or hospitalization (90) and provide information about response to treatment in patients with PAH (88).

### Nitric Oxide

NO is a potent vasodilator produced in ECs by the action of the NOS enzyme in the conversion of L-arginine into L-citrulline and NO (26, 91). In addition to the endothelial NOS (eNOS), there are two other isoforms, the neuronal isoform, and an inducible isoform, which promotes the high diversity of NO biological functions (4). NO can function as a signaling molecule, a toxin, a pro-oxidant, and a potential antioxidant (92). It is involved in the regulation of vascular tone and neurotransmission (93), platelet aggregation, inhibition of VSMCs proliferation (12, 91), destruction of pathogens, and it is a precursor of oxidizing and nitrating species (93). Several stimuli mediate the release of NO, the main stimulus being shear stress (4). NO induces its vasodilatory activity when it is released from the pulmonary vascular endothelium, diffuses through adjacent VSMCs, and stimulates the production of cGMP which, consequently, leads to the activation of cGMP-dependent kinases (91), which in turn increases the myosin light chain phosphatase activity (4), inducing relaxation in VSMCs (4, 12, 91). Given the vasodilator effect of NO and the endothelial dysfunction associated with PAH, it would be expected that patients with PAH would express low levels of NO (30). However, since NO is too unstable to be measured in its gaseous form in the blood, there are several complementary techniques to assess endogenous NO, namely via exhaled NO, nasal NO and nitrate metabolite levels (NOx) in plasma and urine (94). Considering that there are several methodologies to quantify NO levels, we must compare results obtained respecting the same methodology in order to achieve a consistent analysis with the least possible discrepancy.

In PH pathophysiology: several studies showed that exhaled NO levels are reduced in patients with IPAH (94–96). Nevertheless, some studies refute these results, showing that exhaled NO levels may remain similar to healthy patients, or even increase (97–99). Still, there are confounding factors that must always be considered, namely, age, sex, atopy, infections, and medications (100), in addition to methodological differences that may explain the diversity of results (94). The levels of nitrates, nitrites and S-nitrosothiol proteins—products of the NO biochemical reaction -, are in agreement with the levels of exhaled NO, and are also decreased in patients with PAH. Furthermore, NO and oxidative reactions in the lung are correlated with the increase of PAP in PAH (95). Several studies demonstrate that exhaled NO levels increase in PAH patients after treatments with Bosentan (94), and with Epoprostenol—a prostacyclin analog (96, 98). In addition, the use of phosphodiesterase (PDE) inhibitors such as Zaprinast was shown to increases the vasodilator responsiveness to inhaled NO in the lungs of rats challenged with endotoxin (lipopolysaccharide) (101).

Nevertheless, it should be noticed that NO levels are also conditioned by eNOS expression (96). Studies show that there is very little eNOS expression in the vascular endothelium of pulmonary arteries in patients with PH and that the decrease in eNOS expression is inversely correlated with the increase in vascular resistance (102). However, more studies in this sense should be done, since the results are not in agreement; several works assume that there is no expression of eNOS in plexiform lesions (102), while others suggest that there is (103). Hence, since this phenomenon is still not well-understood, the fact of whether there is an increase in the expression of an enzyme does not imply that the enzyme is active (103). Therefore, although there are alterations in the expression of NOS in the pulmonary endothelium, it is not evident that there is a greater sensitivity to the action of NO, as well as a greater activity of this enzyme (4).

Thus, although NO is not functional as a biomarker for PAH, in association with some additional data, it may be useful to understand patients' responses to therapy with prostacyclins (96) and Bosentan (94).

### Asymmetric Dimethylarginine

ADMA is a natural amino acid, derived from the catabolism of proteins containing methylated arginine residues (23, 26). Interest in ADMA has grown over time because it is an endogenous inhibitor of the NOS pathway (104). ADMA was first recognized as a NOS inhibitor in patients with renal failure. In these patients, it was found that ADMA levels increased with the decrease in renal clearance, effects that were reversed with dialysis and restored endothelial function (104). Thus, ADMA is associated with vascular diseases and several risk factors (104) and its plasma levels can perfectly function as a marker of endothelial dysfunction.

In PH pathophysiology: several studies have shown that ADMA plasma levels are increased in patients with PH of different categories including IPAH (11, 105), CHD-PAH (56, 106, 107) and CTEPH (11, 108) and correlate with some hemodynamic parameters. In patients with IPAH, ADMA levels correlated with mPAP, PVR index, SvO_2_, RAP, cardiac index, and survival (11, 105); in patients with CHD-PAH, ADMA levels correlated positively with RAP and negatively with SvO_2_, cardiac output, cardiac index and survival rate (56); and in patients with CTEPH, ADMA levels correlated with mPAP, mRAP, cardiac output, cardiac index, PVR and SvO_2_ (11, 108).

Therefore, circulating levels of ADMA can function as markers of endothelial dysfunction in PAH since the increase in ADMA induces NO synthesis inhibition and consequently will affect the NO/cGMP pathway that is responsible for regulating pulmonary vascular tone, increasing vascular resistance. Some studies also suggested that ADMA can promote pulmonary endothelial dysfunction due to changes in connexin 43 expression and activity, however, the mechanism is not yet known (11). Thus, the fact that ADMA is related to some hemodynamic parameters can be useful to study and monitor the severity of the disease, the effectiveness of the therapies implemented, and assist in risk stratification in patients with PAH or other types of PH (11).

### Cyclic Guanosine Monophosphate

cGMP is the predominant intracellular second messenger of NO. NO activates guanylate cyclase, increasing intracellular cGMP concentrations, thereby inducing vascular smooth muscle relaxation (109). In this way, plasma cGMP would be an alternative marker of eNOS activity (30) since NO inhalation has been shown to induce a significant increase in plasma cGMP levels. On other hand, as cGMP is produced by the activation of the enzyme guanylate cyclase, in addition to being an indirect marker of NO, it will also be an indirect marker of natriuretic peptides, as these are also involved in the activation of guanylate cyclase (110, 111). Furthermore, cGMP has a short life span due to rapid degradation by cyclic nucleotide PDE, with PDE-5 being the most active isoform in lung tissue (112).

In PH pathophysiology: Ghofrani et al. demonstrated that cGMP and ANP levels are high in PAH patients, and that they are closely related to each other and to disease severity (111). In addition to plasma, cGMP can also be measured in urine (30). Bogdan et al. demonstrated that urine cGMP levels are increased in PAH patients and that these levels were higher in patients with severe hemodynamic impairment and may therefore reflect the hemodynamic status of patients with PAH (113). However, besides being related to hemodynamic measures, cGMP is also a relevant therapeutic target, as PDE-5 inhibitor drugs have been increasingly recognized for their action as inhibitors of cGMP degradation. Moreover, the high levels of PDE-5 in the lung VSMCs provide a strong molecular basis for PDE5 inhibitor treatment for PH (114): in patients with PAH, Tadalafil was well-tolerated and improved exercise capacity and quality of life measures and reduced clinical worsening (115); Sildenafil, in an animal model of hypoxia-induced PH, has been shown to reduce PAP, pulmonary vascular muscularization and prevent induced PH (116). Furthermore, Sildenafil proved to be a more effective and selective vasodilator than inhaled NO as it decreases mPAP and PVR and increases the cardiac index, without increasing wedge pressure (117).

Thus, cGMP is a notorious treatment target in PAH, and its plasma and urinary levels can also function as markers of disease severity and hemodynamic impairment.

### D-Dimers

D-dimers are fibrin degradation products, that result from the degradation of the blood clot by plasmin. Each molecule is constituted by two D-domains, linked by disulfide bonds. The detection of D-dimers in the serum is a marker for thrombolytic activity, and this measure is fundamental in the diagnosis of venous thromboembolism—such as pulmonary embolism—and disseminated intravascular coagulation (118, 119). However, D-dimers may also be elevated in other contexts in which there is an activation of the coagulation cascade, such as inflammation, cancer, or pregnancy; furthermore, levels increase with age. Nonetheless, there is a growing interest in the use of D-dimers for diagnostic and prognostic purposes in different clinical settings, like PH.

In PH pathophysiology: PAH pathogenesis involves endothelial dysfunction and *in situ* thrombosis. Moreover, Tournier et al. have described PAH patients with a hypercoagulable phenotype (120). Therefore, considering that D-dimers are products of these phenomenon, they may be useful as diagnostic, prognostic, and therapeutic biomarkers in the context of disease. Shitrit et al. demonstrated that D-dimer levels were elevated in IPAH patients. They also showed that D-dimer levels were correlated with IPAH disease severity, as shown by higher NYHA functional class and mPAP, and lower values of oxygen saturation and 6MWD. Additionally, higher values seemed to predict lower 1-year survival (121, 122). Tournier et al. established that elevated D-dimers in IPAH patients were independent of systemic inflammation (120). However, both studies were conducted with a small number of patients, which limits data interpretation. As for other PAH subtypes, Remková et al. found that elevation of D-dimers in patients with Eisenmenger syndrome (a subtype of CHD-PAH) was non-significant (123). In SSc patients, although D-dimers have been shown to be significantly elevated, no correlation was found with the development of PAH (124), or its severity, as measured by RV systolic pressure (125)—this may be due to a lesser role of microthrombosis in this PAH subtype, or to the low number of patients under investigation.

The main limitation for using D-dimers as PAH biomarkers is that they can be elevated due to inflammatory or pro-coagulatory states of various etiologies, therefore they have very low specificity for diagnostic purposes (119). However, they may be useful for evaluating disease severity.

### Serotonin

Serotonin or 5-hydroxytryptamine (5-HT) is a vasoactive molecule that acts both as a systemic vasodilator and a pulmonary vasoconstrictor. It is produced by enterochromaffin cells, in the gastrointestinal tract, as well as pulmonary neuroepithelial bodies, and then stored in platelets. This storage is dependent on the action of the 5-HT transporter (5-HTT) and results in low plasma levels of 5-HT (10, 19).

In the 1960's, it was suggested that there was an association between the use of appetite-suppressant drugs and PAH, and it was later shown that it was due to interactions with 5-HTT, for which these pharmacological agents act as substrates (126). Since then, new investigations have established that 5-HT promotes pulmonary VSMCs hypertrophy and hyperplasia and pulmonary vasoconstriction, via 5-HTT and serotonin 1B receptor, respectively (1, 10). It can also induce local microthrombosis (127). Moreover, other medications that implicate the serotoninergic system have been shown to increase the risk of developing PAH, such as selective serotonin reuptake inhibitors (17, 128, 129). Regarding 5-HTT, PAH patients have higher transporter expression on vessels and platelets (130), and polymorphisms may implicate disease severity (131–133). Additionally, a study using cultured pulmonary artery cells obtained from PAH patients found that selective 5-HTT inhibitors appeared to have a role in preventing the development of hypoxic PAH, supposedly due to inhibition of the mitogenic response this transporter mediates (130).

In PH pathophysiology: evidence regarding the use of 5-HT as a biomarker for PAH is contradicting. Hervé et al. and Kéreuver et al. both demonstrated that IPAH patients had an increase in plasma serotonin concentrations (and a decrease in platelet levels of 5-HT) (134, 135), that was sustained after heart-lung transplantation, suggesting that higher levels are not secondary to PAH (134). However, 5-HT plasma levels were not predictive of disease severity (135). Later, Zeinali et al. and Lederer et al. failed to find significant different serum measurements of 5-HT between controls and IPAH patients and could not establish a relation between these values and disease severity and 6MWD (136, 137). These discrepancies may be due to a small sample size (the largest study enrolled only 16 patients and 16 controls), different quantification techniques, or even the fact that various PAH subtypes were included in the same samples. 5-HT levels have also been shown to be elevated in PAH associated with ventricular septal defect (138). Recently, Manaud et al. using a rat model of pulmonary veno-occlusive disease induced by mitomycin exposure, demonstrated that pulmonary serotonin level was increased at the very end of pulmonary veno-occlusive disease development, when PH and pulmonary vascular remodeling were already established, indicating that serotonin plays a role late in pathogenesis and/or serves as a marker of PH severity (139).

In conclusion, these findings highlight the potential use of 5-HT as a diagnostic, prognostic, and therapeutic biomarker.

### Osteopontin

Osteopontin (OPN) is a 32-kDa glycoprotein, first described as secreted by malignant epithelial cells (140, 141). It is now recognized to be expressed and secreted by various other cell types, such as cardiomyocytes and fibroblasts, in the context of inflammatory or neoplastic processes (12). It can exist both as a component of the extracellular matrix and a soluble cytokine, acting by increasing cell proliferation, migration, remodeling, and fibrosis (12, 142). Saker et al. found that OPN was one of the most highly expressed matricellular proteins in PASMCs undergoing replicative senescence, and that its release stimulated the migration and proliferation of these cells (143). Later, Mura et al. demonstrated that OPN is among the top five overexpressed genes in transplanted lungs of PAH patients, involved in VSMCs angiogenesis, death, and proliferation pathways; furthermore, its expression was correlated with disease severity (144). Overall, OPN seems to be an important agent in pulmonary vascular remodeling, and, therefore, to the pathophysiology of PAH (145).

In PH pathophysiology: OPN plasma levels were elevated in group I PH, when compared to healthy controls, and predicted all-cause mortality (146, 147). In addition, OPN increased with mRAP, age, 6MWD and NYHA class (146). Rosenberg et al. also found that higher plasma OPN was related to RV remodeling and dysfunction (148). Finally, in the context of IPAH, OPN correlated with NT-proBNP at baseline and during follow-up, providing independent and incremental prognostic information—while NT-proBNP is specific to hemodynamic alterations, OPN may describe the general condition of the patient (148). More recently, OPN plasma levels have been shown to be elevated in other subgroups of PAH, such CTD-PAH (specifically, SSc-PAH) (140) and CHD-PAH (149). In SSc-PAH, OPN was also correlated to patient age (140).

Contrary to all the findings above, a study analyzing group I PH patients irrespective of their disease subtype found that OPN serum measurements conducted at the time of pulmonary artery catheterization did not differ between those and group II PH patients and had no correlation with mPAP (144). As with other biomarkers, the main limitation of these studies is the sample size, that hinders the extrapolation of the findings to other types of PAH. Moreover, the elevation of OPN in both CTEPH (150) as well as PAH (144) leads to the conclusion that this biomarker is most likely related to the development of PH irrespective of its etiology. And, as mentioned earlier, OPN can be produced in the context of other pathologies, such as mesothelioma, breast cancer, or systemic inflammatory disorders, which limits its specificity as a biomarker (140, 148).

### VonWillebrand Factor

Von Willebrand factor (vWF) is a glycoprotein that is produced by the endothelium and acts as a carrier protein for factor VIII. It mediates platelet aggregation and adhesion in response to endothelial activation (12, 26).

In PH pathophysiology: both vWF and its antigen activity (vWF:Ag) have been postulated as group 1 PAH biomarkers. vWF (120, 151–153) and vWF:Ag (154–157) are significantly elevated in primary PAH. It has been shown that elevated baseline vWF significantly predicts short- and long-term survival (151, 155, 158). Functional class and 6MWD were also associated with higher vWF activity; however, in this same study, Al-Naamani et al. determined that lower vWF activity predicts higher risk of death and lung transplant, which is not in concordance with earlier studies (159). Finally, Veyradier et al. and Friedman et al. found lowering in vWF values, parallel with hemodynamic improvements, in PAH patients exposed to prostacyclin treatment (152, 154). In respect to specific PAH etiologies, starting with CHD-PAH, this patient subgroup also showed elevated levels of vWF:Ag (123, 160, 161), and these levels predicted mortality (160, 161). Furthermore, vWF:Ag level may have a role in predicting PAH in SSc, however investigations have not been consensual (162–164). Finally, PAH associated with congenital systemic-pulmonary shunts had significantly raised vWF, that correlated with raises in NT-proBNP (165).

### Microparticles

Microparticles (MPs) are small vesicles formed from membrane blebs (12). They are shed from eukaryotic cells that have been activated or damaged or during apoptosis and, therefore, constitute hallmarks of cell damage. There can be several types, depending on the cell that they derive from: ECs (EMPs), platelets (PMPs) or leukocytes (LMPs) (166) and many authors have postulated their role as biomarkers in cardiovascular diseases (167). Tual-Chalot et al. established a potential role of MPs in PAH pathogenesis (particularly, platelet- and erythrocyte-derived MPs), by determining that these particles acted in reducing eNOS activity and NO bioavailability and increasing production of reactive oxygen species, contributing to dysfunction of pulmonary endothelium (168).

In PH pathophysiology: Bakouboula et al. found that pro-coagulant CD105 or tissue factor positives EMPs were elevated in PAH, and the later subtype correlated with disease severity (6MWD and NYHA class of 3 or more) (169). Similarly, Amabile et al. found heightened levels of various MPs (EMPs - PECAM^+^, VE-cadherin^+^, E-Selectin^+^ - and LMPs) in PAH patients, and significantly correlated PECAM^+^ and VE-cadherin^+^ MPs with hemodynamic severity (170). The investigators then prospectively followed the cohort and observed a significantly higher incidence of negative outcomes (death or decompensated right HF) associated with E-Selectin^+^ MPs, with this biomarker classified as a potential independent predictor of prognosis (171). EMPs can also be found in urine samples, and this marker was elevated in PAH and correlated with tricuspid annular plane systolic excursion, therefore acting as a biomarker for RV function that can be easily measured in a non-invasive manner (172).

Finally, concerning specific PAH subgroups, IPAH was correlated with elevated levels of LMPs—specifically derived from T-cells (173), and PMPs (CD42a and CD42b^+^) (174). Raised EMPs (CD31^+^/CD42b^−^) have been found in both IPAH and SSc-PAH, and variation in measurements taken before and after treatment initiation did not reflect clinical improvement, suggesting this as a marker of endothelial injury (175). Regarding SSc-PAH in particular, elevated VE-cadherin^+^ MPs have also been reported, which measurements appear to independently predict PAH in SSc patients without PH (176). This marker, in addition to CD146^+^ MPs, was also elevated in PAH patients with Eisenmenger Syndrome (177).

Despite significant advancements in this field of research in recent years, one of the main challenges we are now facing regards quantification and phenotyping of MPs. Flow cytometry surpasses previous techniques, such as annexin-V labeling (167), being able to both detect and determine cellular origin of MPs. Moreover, it can be more easily scaled up, allowing for the study of large patient cohorts (172). However, even the most sophisticated equipment available is limited in the particle size range it can detect, with most MPs being under the minimum size limit (178). This hinders the reliability of new findings. Still, MPs arise as a promising biomarker for disease severity and prognosis in PAH.

## Biomarkers of Angiogenesis

### Angiopoietins

Angiopoietins, in particular angiopoietin-1 and 2 (Ang-1 and Ang 2, respectively), are angiogenic factors, produced by SMCs and precursor pericytes to regulate the development of lung vasculature (12, 19). Their actions are mediated via Tie-2 receptor, with Ang-2 acting as an antagonist to Ang-1 (12). It has been difficult to establish the role of angiopoietins in PAH, but it is now widely recognized that the Ang-Tie2 signaling pathway plays a major role in PAH pathogenesis, through the regulation of vascular hyperplasia (12). However, tonic pathway activation appears to also play a protective role. On one hand, Tie-2 stimulation leads to inhibition of *BMPR2* signaling—the gene that is altered in hereditary cases of PAH—and increase in production of serotonin by pulmonary arteriolar ECs. These changes concur to promote PAH. On the other hand, the Tie-2 receptor pathway can downregulate endothelial activation and remodeling, therefore mitigating its deleterious effect (19, 179). Moreover, Ang-2 expression has been found to be up-regulated in plexiform lesions from lung tissue samples (180).

In PH pathophysiology: Kumpers et al. determined that, while venous plasma levels of Ang-1 and Ang-2 were elevated in IPAH patients, only Ang-2 correlated with mRAP, PVR, NYHA functional class, cardiac index and SvO_2_. Ang-2 also significantly predicted mortality, and values after initiating treatment varied inversely to changes in mRAP, PVR, and SvO_2_ (180). Another study showed that Ang-2 was elevated in IPAH patients, and its decrease reflected improvements in 6MWD during Treprostinil monotherapy (181). Recently, Ang-2 was also shown to be elevated in SSc patients that develop PAH, but studies regarding correlation with disease severity have yet to be conducted (182). As for Ang-1, significantly different levels between PAH patients and healthy individuals have been inconsistently found; and there was no association with clinical or laboratory parameters (180, 183).

Therefore, Ang-2 independently predicts disease survival, severity, and response to treatment, and appears to outperform NT-proBNP in doing so, and it is equally easy to determine (180).

### Vascular Endothelial Growth Factor

Vascular endothelial growth factor (VEGF) is an angiogenic factor. It has several isoforms which act as ligands to specific receptors. Regarding PAH, the isoforms VEGF-A and -B and the receptors VEGFR-1 and−2 have been most extensively researched and linked to PH pathogenesis (19). They have been traditionally regarded as promoters of angiogenesis deregulation (184, 185), but recent investigations postulate a protective role against these pathologic alterations (186).

In PH Pathophysiology: VEGFR expression is increased in IPAH, with VEGFR-2 being found mostly overexpressed in plexiform lesions (184, 185). VEGFR-2 is lower in PAH (187), whereas soluble VEGFR-1 (sVEGFR-1) is elevated (188–190). sVEGFR-1 is the most well-researched marker, and it has been shown to predict functional class, disease severity (188, 190), patient survival (188), and adverse events (191). Particularly, Kylhammar et al. studied sVEGFR-1 in SSc-PAH and IPAH and found that both patient groups had significantly elevated values of this marker; additionally, sVEGFR-1 was able to predict treatment response, and was higher is SSc patients that would later develop PAH (189). This later finding is supported by other studies (192). Finally, it has been shown that sVEGFR-1 correlates to RV systolic pressure and the capacity of diffusing carbon monoxide in SSc-PAH (192); therefore, sVEGFR-1 arises as a potential non-invasive screening tool for SSc-PAH (190).

Regarding the VEGFR ligands, they have been documented as significantly increased in various forms of PAH, namely IPAH (180, 187, 189, 193) and SSc-PAH (162, 189, 194). Contrary to this, a 2014 systematic review found that, although serum VEGF levels were not increased and did not correlate with hemodynamic alterations, VEGF expression on arterial vascular ECs was significantly higher in CHD-PAH and IPAH patients when compared with healthy controls; additionally, higher expression predicted worse outcomes post-surgical treatment of the patients with CHD (106).

### Endoglin

Endoglin (Eng), also known as CD105, is an anti-angiogenic agent that exists in two forms, soluble (sEng) and membrane bound. It is expressed in proliferating ECs and has an important role in vascular development and pathogenesis of vascular diseases, such as pre-eclampsia and tumor angiogenesis. Eng acts as an auxiliary receptor for transforming growth factor-β (TGF-β), modulating several signaling pathways, and its overexpression is associated with dysregulated angiogenesis (195)—in PAH, enhanced Eng expression has been found in plexiform lesions, which is in favor of Eng as an indicator of vascular proliferation and remodeling (188).

In PH pathophysiology: sEng was elevated in PAH patients and had higher sensitivity for the presence of PAH than NT-proBNP. Likewise, sEng was predictive of NYHA functional class and performed significantly better than NT-proBNP, being sensitive even among mildly symptomatic patients (NYHA Class I-II); moreover, an equally weighted combination of sEng and NT-proBNP was not significantly better than sEng alone. Finally, sEng independently predicted patient survival (188). Another study detected significantly raised levels of sEng on SSc-PAH patients *vs*. healthy controls (196).

Overall, sEng arises as a biomarker that appears to be more sensitive for risk stratification than NT-proBNP. Moreover, it is not influenced by RV dysfunction, making it more specific (188). Finally, it could detect PAH even in patients with minimal symptoms. Therefore, Eng is a promising diagnostic and prognostic biomarker and deserves further studies to validate these findings.

### Bone Morphogenic Protein 9

Bone morphogenic protein 9 (BMP9), also known as Growth Differentiation Factor 2, is a liver derived protein present in peripheral circulation, belonging to the TGF-β superfamily (197, 198). It can act as a hematopoietic (199), hepatogenic (198), osteogenic (200), or chondrogenic factor (201), and several BMP9 genetic variants have been associated with different pathological states (197).

In PH pathophysiology: BMP9 is implicated in the BMP9/*BMPR2*/endoglin/ALK1 signaling axis - it is a physiological ligand to the *BMPR2*/ALK1 receptor complex, and to coreceptor endoglin—acting as a circulating factor for maintenance of vascular quiescence, by inhibiting EC migration and growth in the pulmonary vasculature (202–204). Mutations of the *BMP9* gene and its receptor and coreceptor are among the most common mutations present in hereditary cases of PAH (205–208) and several pathogenic *BMP9* genetic variants have also been found IPAH patients (209–211). Moreover, in IPAH patients, median plasma levels of BMP9 have been found to be significantly lower than those of control patients (209, 210, 212), with even lower plasmatic levels and plasma activity in patients carrying certain BMP9 mutations (209, 211).

In recent years, research efforts have been focused on the implications of BMP9 levels and genetic variants in patients with PAH associated with severe liver disease—portopulmonary hypertension (PoPH). Nikolic et al. studied BMP9 plasma levels in patients with PAH of groups 1, 2 and 3, focusing particularly PoPH, and found markedly lower BMP9 levels in PoPH patients *vs*. healthy controls, *vs*. PAH of other etiologies, and *vs*. group 2 and 3 PH (213). Moreover, BMP9 was able to distinguish PoPH from liver disease without PAH in humans and rat models, suggesting a potential diagnostic application; and was predictive of transplant-free survival in all patients with group 1 PAH, therefore also implicating disease prognosis. No correlation with measures of RV function and disease severity was found. The same research group studied rat models of PAH with portal hypertension and cirrhosis and described an exacerbation of PH phenotype and pulmonary vascular remodeling upon administration of a BMP9 ligand trap, highlighting an apparent protective effect of endogenous BMP9 (213). These findings are supported by those of Long et al., who found that administration of exogenous BMP9 ameliorated PH induced by toxin exposure or *BMPR2* mutation, as shown by improvements in vascular remodeling and RV hypertrophy in several animal models (214). Contrary to these findings, a recent investigation by Tu et al. associated the loss of BMP9, either by deletion or inhibition, with protection against hypoxia and monocrotaline induced PAH; additionally, they described lower mRNA levels of ET-1, and higher levels of ADM, translating a de-regulation of endogenous vasoactive agents (215).

These opposing findings give insight into the complexity of BMP9 signaling and its potential implications in several mechanisms underlying PAH pathophysiology. In addition, they highlight the many potential clinical uses of BMP9, either as a diagnostic and/or prognostic biomarker, or as a novel therapeutic target. Regarding PoPH and considering the high fatality rate associated with this PAH subtype (216), BMP9 may constitute an important tool for clinical screening of high-risk populations; nonetheless, more studies evaluating biomarker performance are still needed to validate these new concepts.

### Endostatin

Endostatin (Es) is an angiostatic peptide that results from the cleavage of collagen XVIII, which exists on the extracellular matrix, predominantly that of the vasculature (217). It is known as an inhibitor of endothelial proliferation, angiogenesis, and tumor growth (218). The action mechanism via which it intervenes in PAH has yet to be determined; however, recent findings showed that Es inhibits pulmonary artery ECs proliferation and migration and promotes ECs apoptosis, in line with PAH pathogenesis (219).

In PH pathophysiology: Es has been found to be elevated in PAH, particularly in IPAH (220, 221), CTD-PAH (221) and CHD-PAH (222). In IPAH, elevated Es levels correlated with unfavorable hemodynamic alterations (higher mPAP and PVR, lower cardiac index and cardiac output), worse functional class, and reduced exercise tolerance (220, 223). Moreover, Es strongly predicted disease mortality in this disease group (223). Similarly, regarding IPAH and CTD-PAH, Simpson et al. determined that the elevation of Es on patient blood samples independently predicted disease severity (measured by mRAP, mPAP, PVR, 6MWD, pulmonary artery compliance and stroke volume) and mortality—this association was particularly strong in IPAH (221). Considering the case of SSc, Es appeared to predict the development of PAH in these patients (224). Thirdly, Daly et al. investigated Es in CHD-PAH and found that, besides predicting worse hemodynamics and functional capacity, Es elevation was directly correlated to several echocardiographic alterations, predicting RV dysfunction; also, its levels lowered as patients showed clinical and hemodynamic improvement (222). Lastly, Es has been tested has a tool to predict clinical outcomes in IPAH, and CHD and CTD-PAH, in addition to tools that are currently validated [REVEAL, ESC/ERS (221), and NT-proBNP (222)], and it was able to improve their performance in risk discrimination and mortality stratification.

Es arises as a robust prognostic biomarker and a potential candidate for the update and refinement of current risk assessment strategies. Additionally, investigation into the pathobiology of Es in PAH is also needed—should it prove to be an agent in PAH pathophysiology, that would wield it a degree of specificity surpassing that of NT-proBNP.

## Biomarkers of Inflammation/Oxidative Stress

### C-Reactive Protein

CRP belongs to a family of highly conserved proteins, the pentraxins (225). CRP is predominantly synthesized in hepatocytes in response to cytokines such as interleukin (IL)-6 and IL-1, functioning as a representative of the acute state (225, 226) and as a sensitive marker of underlying systemic inflammation (227). However, during inflammation, CRP can be produced in different forms (225). In addition to being prevalent in inflammation, CRP also plays a key role in endothelial dysfunction (227), atherosclerosis and cardiovascular diseases, having been recognized as a risk predictor of cardiovascular diseases (225, 226, 228) and a risk predictor of pulmonary arterial diseases (229).

In PH pathophysiology: studies showed that CRP levels are increased in PAH patients (227, 228). There is evidence that CRP induces the production of IL-6 and monocyte chemoattracting protein-1 (MCP-1), known systemic inflammatory markers, both associated with the development of PAH in animal models (229). This was corroborated by Li et al., which showed that these pro-inflammatory agents are also increased in cultured VSMCs from PAH patients (227). Li et al. suggest that CRP regulates the expression of IL-6 and MCP-1 in VSMCs by nuclear factor kappa B (NF-κB) pathway (227). NF-kB is a crucial transcription factor in the inflammatory response, involved in the transcription of several cytokines, chemokines, and adhesion molecules. Furthermore, it appears to be involved in the development of PAH: in an animal model of PAH, the administration of a NF-kB inhibitor improves PH manifestations (230). Moreover, it was found that treatment with atorvastatin has an anti-inflammatory action associated with the NF-kB pathway, decreasing the levels of MCP-1 and IL-6 induced by CRP in a dose-dependent manner (227). Quarck et al. showed that in addition to CRP levels being higher in patients with PAH, these correlate with RAP, NYHA functional class, 6MWD and survival, and predict outcome and response to therapy (228). Furthermore, Quarck et al. demonstrated that in PAH patients whose treatments are effective and stabilize plasma CRP levels, the survival rate is significantly higher, accompanied by a decrease in NYHA functional class and an increase in cardiac index (228). In CTEPH, plasma CRP levels decrease significantly after surgery (pulmonary endarterectomy) (228).

Therefore, this evidence shows that CRP can function as a biomarker of PAH, suggesting the inflammatory status of these patients and guiding the level of therapeutic options.

### Growth Differentiation Factor-15

Growth differentiation factor-15 (GDF-15) or macrophage inhibiting cytokine is a stress-responsive member of TGF-β cytokine superfamily (231). GDF-15 is closely involved in tissue differentiation, remodeling, and repair (232), and it is strongly expressed in activated macrophages and epithelial cells (233). Under normal conditions, GDF-15 is poorly expressed in tissues, however, in pathological conditions—acute injury, tissue hypoxia, inflammation or oxidative stress—its expression is significantly increased (234). At the heart level, in normal situations, the myocardium does not express GDF-15, which increases dramatically after pressure overload or myocardial ischemia (235). Studies showed that circulating levels of GDF-15 are increased in patients with cardiovascular diseases, namely with chronic HF (236) and acute coronary syndrome (237), proving GDF-15 levels provide prognostic information and function as a biomarker of risk of death in these patients (236, 237). Lankeit et al. also showed that GDF-15 levels are increased in patients with pulmonary embolism and that these are related to the increased risk of death and major complications in the first 30 days after diagnosis. Furthermore, the prognostic information of GDF-15 is complementary to that of the biomarkers NT-proBNP, cTnT and the echocardiographic findings of RV dysfunction (235).

In PH pathophysiology: Nickel et al. showed that GDF-15 levels are increased in patients with IPAH, being closely related to more severe disease and poor prognosis in these patients (234). In addition, it was found that GDF-15 levels do not correlate with hemodynamic parameters and cannot be used as a diagnostic marker for PH, but, on the other hand, they do correlate with baseline NT-proBNP levels (234), which is in line with what was observed in patients with chronic HF (236) and acute pulmonary embolism (235). Moreover, measuring GDF-15 levels in combination with NT-proBNP achieved an improvement in the detection of high-risk cases (234). Nickel et al. also demonstrated that the expression of GDF-15 is increased in the lungs of patients with PAH and that GDF-15 is predominantly located in vascular ECs and the center of plexiform lesions (238). Therefore, GDF-15 arises as a potential marker for disease severity that correlates with NT-proBNP measures and can improve the prognostic performance of this validated biomarker.

### Uric Acid

Serum uric acid (UA) is the end-product of adenine oxidation and guanine purine metabolism (239, 240). When this oxidative metabolism is impaired, there is an increase in UA levels due to the decrease/depletion of ATP levels, promoting the catabolism of adenine nucleotides into inosine, hypoxanthine, xanthine and UA, by increasing the expression of the xanthine enzyme oxidase (239, 240). In situations of hypoxia, ischemia, and some pathologies, such as chronic HF, cyanotic congenital heart disease, and COPD, UA levels are increased as a reflection of compromised oxidative metabolism (240, 241). Hyperuricemia is closely correlated with symptom severity and high mortality in patients with chronic HF, and serum UA may function as an independent marker of impaired prognosis in these patients (241, 242).

In PH pathophysiology: Nagaya et al. demonstrated that PAH patients have increased levels of UA compared to controls. Furthermore, serum UA levels positively correlate with more severe NYHA functional class, total pulmonary resistance, and mortality, and negatively correlate with cardiac output (240). Also, an approach with vasodilator therapy promotes a decrease in UA levels, associated with a reduction in total pulmonary resistance (240). Additionally, other studies corroborate these results and effectively show that serum UA levels are elevated in patients with PAH and correlate positively with NYHA functional class and mortality and negatively with the 6MWD (243). Recent studies showed that high serum UA levels are associated with a poor prognosis at first follow-up. Furthermore, the increased levels of UA promote a slight increase in the growth of PASMCs in IPAH patients when compared to controls (244). Thus, UA levels are elevated and correlated with disease severity in PAH (239, 240, 243), can be used as a non-invasive clinical prognostic indicator during follow-up (244), and may even function as a therapeutic marker.

The use of serum UA in combination with other biomarkers greatly increases the possibilities of diagnosis, so its use should be combined with more markers (245). However, it should always be considered that serum UA levels are affected by renal activity since approximately two-thirds of UA is excreted through the kidneys and one third through the gastrointestinal tract. Therefore, patients with renal failure or patients recommended for diuretic therapy should not be considered as their serum UA levels may be misleading (239). In addition, some hormonal factors and/or circulating substances (such as catecholamines, angiotensin II, ET, thromboxane, ANP) may influence impaired renal UA removal (239) and therefore interpretation may be difficult in some patients.

### Monocyte Chemoattracting Protein-1

MCP-1 is one of the main pro-inflammatory chemokines, which are a family of chemo-attracting cytokines, subdivided into four families (246). MCP-1 has a strong chemoattractant activity for monocytes and macrophages, being responsible for the regulation/activation and infiltration of monocytes and macrophages (246), leading to the induction of cytokine secretion and expression of adhesion molecules (247). MCP-1 is essentially produced in response to inflammation, and it is synthesized by several cells, including monocytes/macrophages, vascular ECs, VSMCs and fibroblasts (227, 247). In addition, MCP-1 is also released by pulmonary artery ECs, having a direct role in the infiltration of monocytes in the injured vessel wall, as well as in the proliferation of PASMCs (227).

In PH pathophysiology: Sanchez et al. demonstrated that MCP-1 overproduction may be a feature of the abnormal pulmonary ECs phenotype in IPAH, contributing to the inflammatory process and to pulmonary vascular remodeling (248). Previous studies showed that plasma MCP-1 levels are elevated in patients with CTD-PAH (247). Hashimoto et al. showed that serum MCP-1 levels are elevated in patients with IPAH and that there is a response to Epoprostenol therapy (249). Itoh et al. also showed that MCP-1 plasma levels are elevated in patients with IPAH, and this elevation was particularly marked in the early stage of disease. However, they do not correlate with the duration of the disease, nor do they correlate significantly with the hemodynamic variables (247), which was verified by Hashimoto et al. (249). Moreover, studies in rats with monocrotaline induced PH show that inhibition of MCP-1 signaling inhibits the increase of MCP-1 levels as well as pulmonary vascular remodeling, improving disease prognosis (250). Thus, although MCP-1 does not correlate with hemodynamic variables, it should be noticed that it contributes to the development of PH. Thus, the assessment of MCP-1 levels can become a useful tool in the early diagnosis of PH (247).

### Galectin-3

Galectin 3 (Gal-3) is a member of the lectin family and of the β-galactoside-binding protein family (251, 252). It is expressed in the nucleus, cell surface and extracellular space and it is also expressed in inflammatory cells, fibroblasts, and myocardium (251, 252). Gal-3 binds to several substrates, which include signaling molecules, transcriptional regulators, ribonucleoproteins, cell surface receptors and matrix proteins and, for this reason, plays a fundamental role in several biological functions, namely, in proliferation, migration, adhesion, differentiation, angiogenesis, inflammation, apoptosis and fibrosis (251). That is why it is involved in remodeling and vascular stiffness (253). Gal-3 has pathogenic activity at the level of cancer, inflammatory and fibroproliferative diseases, such as pulmonary, cardiac, and hepatic fibrosis, and therefore, depending on the disease, Gal-3 is increased in different types of cells, which include macrophages, fibroblasts, and carcinogenic cells (251). Gal-3 is a well-known biomarker of fibrosis and of chronic left ventricular HF (254, 255). When expressed by activated macrophages and ECs, Gal-3 activates an inflammatory response and extracellular matrix remodeling (254, 255), inducing fibroblast proliferation and collagen synthesis, which contribute to cardiac remodeling, a determining factor in the development and progression of HF (251).

In PH pathophysiology: several studies show that Gal-3 serum levels are increased in patients with PAH (256), which may explain the development of vascular and RV fibrosis (254, 255). A systematic analysis of blood cells mRNA profiles from pre-transplant patients of the lung showed that the gene that most contributes to PAH, according to the Prediction Analysis of Microarrays, was Gal-3 (*LGALS3*). Gal-3 seems to be involved in RV HF, one of the most common causes of death in PAH patients. Moreover, this over-expression was validated in an independent PAH group by qPCR (257). Fenster et al. argue that, possibly, Gal-3 is released by the RV myocardial macrophages in response to changes in pulmonary artery pressure and volume. In addition, the levels of tissue inhibitor of metalloproteinase-1, a marker of myocardial matrix renewal, were increased in serum of PAH patients and were positively correlated with Gal-3; they may reflect the metabolism status of the RV myocardial extracellular matrix (256). On the other hand, Mazurek et al. also showed that Gal-3 levels are high in patients with PAH but that there is no correlation between Gal-3 levels and the structural and functional parameters of RV. They further argue that increasing Gal-3 levels are strongly predictive of mortality in any etiology of PH, including PAH. It should be noticed that all these studies are limited by the small sample size (256, 258). Furthermore, Scelsi et al. showed that plasma levels of Gal-3 increase linearly in the five risk strata on the REVEAL 2.0 risk scale. Furthermore, plasma levels of Gal-3 are also increased in patients who have a greater impairment of RV performance (259).

We can conclude that plasma levels of Gal-3 are associated with the various risk profiles of PAH (259). However, although Gal-3 has the potential to be a PAH biomarker, there are some confounding factors such as age, sex, diabetes, systemic hypertension, body mass index, BNP and NT-ProBNP (260). In addition, Gal-3 can be used as a biomarker of renal failure and it is present in pulmonary and hepatic fibrosis, making it useful as a specific biomarker of PAH only when these comorbidities are not present (256).

### Interleukins

Inflammation is a characteristic of PAH, with high levels of circulating cytokines in patients with PAH (261). Several studies show the role of inflammatory cytokines in the development of IPAH; in addition, some animal studies also support the role of inflammatory cytokines in the initiation and progression of PAH (261). IL-1β, IL-6 and tumor necrosis factor- α (TNF- α) are pro-inflammatory cytokines, produced by monocytes, macrophages, and ECs (252). Additionally, they can induce the proliferation of fibroblasts and SMCs and promote thrombosis. Thus, they have a preponderant role in the initiation and progression of the growth of SMCs, fibroblasts and ECs and the occurrence of microthrombotic lesions commonly associated with severe PH (252).

In PH pathophysiology: serum levels of IL-1β, IL-6 and TNF-α are increased in patients with PAH (252, 262). Humbert et al. showed that IL-6 levels had an impact on the survival of patients with PAH; however, they did not correlate with hemodynamic parameters (252). Consistently with these results, Soon et al. showed that other cytokines are increased in circulation, namely, IL-2, IL-4, IL-8, IL-10, and IL-12p70. Thus, it can be concluded that there is a deregulation of the circulating cytokines in PAH patients. Additionally, it also showed that the levels of IL-2, IL-6, IL-8, IL-10, and IL-12p70 are predictors of survival in these patients (261). Therefore, serum levels of cytokines are more correlated with patient survival than with the indexes of RV function; there was no correlation between cytokines and hemodynamic parameters, which may mean that cytokines are associated with the pathogenesis of PAH and not only with RV function (261).

The levels of IL-1β and IL-6 are clearly increased after exposure to hypoxia, and this increase is closely related to the activation of the immune system, responsible for inflammatory activity (263). However, the molecular mechanism involved is still not well-established and, therefore, several animal studies have been carried out in this direction. Savale et al. demonstrated that IL-6 appears to have a preponderant role in the modulation of inflammation, vascular remodeling, and the development of PH, presenting an exaggerated response to hypoxia. In studies with IL-6-deficient mice (IL-6^−/−^), there was a clear attenuation of PH and RV hypertrophy; even when IL-6^−/−^ mice were exposed to hypoxia, they presented lower recruitment of inflammatory cells in the lungs than wild-type mice (264). On the other hand, Hashimoto-Kataoka et al. identified IL-21 as a target downstream of IL-6 signaling in PAH, suggesting that the IL-6/IL-21 signaling axis is involved in the pathogenesis of PAH, together with an accumulation of M2 macrophages in the lungs. In addition, they also found an increase in IL-21 expression and M2 macrophage markers in lung samples from patients with IPAH (265). Regarding IL-1β, Parpaleix et al. suggest that binding of IL-1β to its receptor, IL-1 receptor1 (IL-1R1), promotes recruitment to the primary adapter myeloid differentiation protein of molecular adapter 88 (MyD88) and induces the synthesis of IL-1, IL-6, and TNF-α through NF-κB activation. This pathway appears to play a major role in the pathogenesis of PH, affecting the proliferation of VSMCs and the recruitment of macrophages. In addition, patients with IPAH and mice with hypoxia-induced PH have an increased expression of IL-1R1 and MyD88 in the pulmonary vessels. Moreover, MyD88-deficient mice (MyD88^−/−^) showed attenuation in PH, which suggests effects mediated by IL-1β in PASMCs and macrophages. Therefore, the IL-1R1/MyD88 signaling axis is closely involved in the remodeling and inflammation of the pulmonary vessels (263).

Thus, we can conclude that, although cytokines do not stand out for the role of PAH biomarkers, it is possible to establish a relationship between some cytokines and the survival of patients with PAH. Additionally, Duncan et al. showed that there is an increase in levels of circulating cytokines and growth factors and their correlation with outcome in pediatric PAH (191). Some pathways could be potential targets of study in the treatment of PAH, namely, the pathways IL-6/IL-21 (265) and IL-1β/IL-1R1 (263).

### Isoprostanes

Isoprostanes are formed when reactive oxygen species such as peroxide, superoxide and peroxynitrite react with unsaturated membrane lipids, such as arachidonic acid (92, 266) (molecules with a cyclopentane ring and two cis alkyl chains between them), isomers of prostanoids (with two trans chains between them) (267). Isoprostanes are essentially biomarkers of oxidative stress, however, they have several biological effects (267). F2-isoprostanes (F2-isoP) are one of the most stable biomarkers of lipid peroxidation when measured in urine (267, 268). 15-F2t-isoprostane (15-F2t-IsoP), or 8-iso-prostaglandin F2, in one of the most abundant isoprostane *in vivo* (268) and can be measured in urine and plasma (267). 15-F2t-IsoP has potent vasoconstrictor activity—it is involved in vasoconstriction of pulmonary arteries and resistance microvessels—and mitogenic activity in VSMCs (267). In addition, 15-F2t-IsoP stimulates ECs proliferation and ET-1 synthesis in pulmonary arteries (269).

In PH pathophysiology: studies suggest that F2-isoP may function as a marker in the early stages of PAH (270) as F2-isoP is increased in both urine (271, 272) and plasma (272) in different subgroups of PAH (including IPAH, hereditary PAH and asymptomatic patients predisposed to PAH because of *BMPR2* loss-of-function mutations) (273). Increased plasma levels of 15-F2t-IsoP in patients with IPAH correlate with WHO functional class severity, lower 6MWD and SvO_2_, and higher mRAP and BNP levels (272). Additionally, studies demonstrated that increased levels of urinary F2-isoP are inversely correlated with pulmonary vasoreactivity (274) and that urine 15-F2-isoP levels, when quantified during initial diagnosis, were independently associated with an increased hazard of death in a cohort of patients with PAH (268). These results suggest that urinary F2-isoP may represent a biomarker with prognostic potential in PAH.

### Oxidized Lipids

The role of oxidized fatty acids and oxidized phospholipids in atherosclerosis and other inflammatory diseases is well-established (275, 276). Biological metabolites of arachidonic and linoleic acid, hydroxyeicosatetraenoic acids (HETEs), and hydroxyoctadecadienoic acids (HODEs), respectively, play a critical role in the pathogenesis of atherosclerosis (275, 276).

In PH pathophysiology: concentrations of oxidized fatty acids- 5-, 12-, and 15-HETE, and 9- and 13-HODE - are increased in the plasma and in lung tissues of patients with PAH (277–279) and in several animal models of PH (280, 281). Increased 15-HETE levels in the context of PH have been shown to induce VSMCs pro-proliferative/anti apoptotic phenotype (282), inflammation (277), and fibrosis (283). Recently, Ruffenach et al. (284) demonstrated that dietary 15-HETE is sufficient to induce PH in mice. In this study, unbiased large-scale transcriptomics identified key pathways that are dysregulated by dietary 15-HETE and further confirmed the dysregulation of similar pathways in patients with PAH. They established that increased ECs apoptosis by 15-HETE via a T cell–dependent mechanism is one of the mechanisms triggering PH in mice. They also demonstrated that the apolipoprotein A-I mimetic peptide Tg6F (transgenic 6F), which has previously been shown to reduce plasma oxidized lipids and atherosclerosis (285, 286), can prevent and rescue PH development in mice.

### CD40/CD49L

CD40 is a type I transmembrane receptor belonging to the TNF superfamily of receptors. Its ligand (CD40L) and its soluble form (sCD40L) have immunomodulating activity and likewise belong to the TNF superfamily (287). CD40 is expressed primarily on B cells, but also on other cells of the immune system (macrophages, basophils, T cells), on epithelial cells, fibroblasts, ECs, VSMCs, platelets and dendritic cells (287). Thus, the interaction of CD40 with CD40L, in its soluble and/or transmembrane form, induces the activation of inflammatory and coagulatory pathways of the vascular endothelium and promotes the activation of immune and non-immune cells (288), triggering an inflammatory response, degradation of the matrix and formation of thrombi (289).

In PH pathophysiology: Damås et al. demonstrated that patients with PAH have increased levels of sCD40L. They also suggested that the CD40-CD40L interaction contributes to the increased expression of chemokines in patients with PAH: recombinant sCD40L induced the production of IL-8 and MCP-1 in ECs, and plasma levels of these chemokines increased in PAH patients, significantly correlating with sCD40L and hemodynamic parameters. Furthermore, platelets from patients with PAH showed greater release of sCD40L compared to the control group (289). Another study suggests that endothelial progenitor cell transplantation, a novel and experimental therapeutic option for PAH, could yield higher efficacy when turning off the CD40 pathway is turned off in the transplanted cells may have advantages in therapies used to treat PAH, namely endothelial progenitor cell transplantation (288).

## Metabolic Biomarkers

### Tryptophan Metabolites

Tryptophane is an essential amino acid that can be metabolized through two different enzymatic pathways. The tryptophane hydroxylase pathway results in the conversion of tryptophane to serotonin, whereas the indoleamine 2, 3-dioxygenase (IDO) pathway can produce various tryptophan metabolites (TMs), such as kynurenine, kynurenate, anthranilate and quinolinate (290, 291).

In PH pathophysiology: a recent study conducted on rats with monocrotaline induced PAH found that TMs pathways, among others, are dysregulated in PAH, due to probable metabolic reprograming events in disease pathogenesis (292). IDO-TMs, and not tryptophane hydroxylase metabolites, were elevated in PAH and strongly correlated with RV-pulmonary vasculature dysfunction (resting RAP, PAP, PVR, exercise PVR and change in cardiac output during exercise) in a cohort of patients with PAH (291). Moreover, the predominant IDO-TM, kynurenine, has been more thoroughly investigated and, aside from being significantly higher in PAH patients (293–295), it correlated with mPAP and PVR (293) and could predict negative patient outcomes (294).

### Ghrelin

Ghrelin is traditionally viewed as a “hunger hormone,” produced in the gastrointestinal tract. It acts as an endogenous ligand for growth hormone secretagogue receptors and can regulate energy metabolism, glucose metabolism, gastrointestinal motility, and food intake. However, it is also involved in modulating cardiovascular function, playing a role in some cardiovascular diseases (296) and has been shown to be a physiological antagonist of ET-1 mediated vasoconstriction *in vitro* (297). In the serum, ghrelin can be found as acyl-ghrelin or des-acyl ghrelin, with the latter constituting the larger but inactive fraction (298).

In PH pathophysiology: in IPAH, total ghrelin plasma levels were elevated and predicted RV hemodynamics (RV diameter and pulmonary artery systolic pressure) (299). As for CHD-PAH, acyl-ghrelin was raised, and correlated, additionally, with pulmonary artery diastolic pressure, RV systolic pressure, mPAP, and pulmonary artery trunk diameter (300). Lastly, ghrelin levels correlated with N-BNP (299), ET-1 and NO measures (299, 300). In atrial septal defect patients with PAH, ghrelin is increased and correlates negatively with mPAP, which can suggest that ghrelin levels can predict the severity of PH in patients with atrial septal defect and PAH (301).

Furthermore, studies in animal models of PH showed that ghrelin can modulate PH: in chronically hypoxic rats, exogenous administration of ghrelin attenuated the development of PH, pulmonary vascular remodeling, RV hypertrophy, and overexpression of eNOs and ET-1 (302); also, in rats with PH induced by monocrotaline, exogenous administration of ghrelin attenuated PH, RV hypertrophy, wall thickening of peripheral pulmonary arteries, and RV diastolic disturbances and ameliorated left ventricle dysfunction, without affecting its endogenous production (303). This highlights a potential role for ghrelin a new therapeutic weapon against PAH.

## Transcriptional Regulators and Oncogenes Expression

### PIM-1

PIM-1 (Moloney Murine Leukemia Provirus Integration Site) is a proto-oncogene that encodes a serine/threonine kinase (304, 305). Its expression is induced by a variety of cytokines, growth factors, and mitogens (304). PIM-1 is closely related to cell cycle regulation, being involved in cell proliferation and survival (304), and overexpression of PIM-1 is related to the development and progression of various cancers, by increasing cell proliferation and resistance to apoptosis (306).

In PH pathophysiology: PIM-1 has been associated with the development of PAH, becoming a potential PAH biomarker. Katakami et al. demonstrated that PIM-1 is involved in VSMCs proliferation and neointima formation when associated with arterial wall lesions; that is, lesions in the arterial wall promote the release of mitogens and the induction of proto-oncogenes, which, in turn, will promote the proliferation of VSMCs and neointimal formation. Furthermore, they suggest that PIM-1 expression in cultured VSMCs is markedly induced by oxidative stress (304). Although the mechanism of action of PIM-1 in VSMCs proliferation is not totally understood, it is known that PIM-1 phosphorylates and activates Cdc25A, a phosphatase that promotes cell cycle progression, and c-Myb, a transcription factor that is essential for VSMCs replication (304). In addition, it cooperates with c-Myc, which plays an important role in VSMCs proliferation (304, 305). Moreover, studies demonstrated that PIM-1 contributes to the activation of the nuclear factor of activated T cells (NFAT)/signal transducers and activators of transcription-3 (STAT3) signaling pathway (306), which is a pathway highly involved in pro-proliferative and anti-apoptotic phenotype of VSMCs in PAH (307, 308). Paulin et al. showed that PIM-1 is increased in PAH, both in experimental studies and in patients with PAH, and that it is dependent on STAT3 activation. In addition, PIM-1 expression parallels NFAT activation and pulmonary artery remodeling and pressure and correlates with disease severity, suggesting that NFAT activation via PIM-1 is specific to pulmonary vascular remodeling (306). STAT3 and NFAT are expressed in several tissues, including the pulmonary artery, contrary to PIM-1, which has a lower expression in healthy tissues, and it is essentially expressed in PAH-PASMCs (306). Renard et al. studied the levels of PIM-1 in patients with PAH (IPAH, CTD-PAH, vasoreactive-IPAH and CHD-PAH) and verified that the levels of PIM-1 in plasma are increased in patients with IPAH and CTD-PAH, patients essentially characterized by active pulmonary vascular remodeling. On the other hand, patients with vasoreactive-IPAH and CHD-PAH had normal plasma levels of PIM-1, suggesting that vascular remodeling via the STAT-3/NFAT/PIM-1 pathway is limited in some PAH phenotypes. In addition, it was found that among PAH patients, PIM-1 levels correlated with traditional markers of disease severity and predicted mortality (309), which makes PIM-1 a potential biomarker for PAH, essentially as a representative biomarker of pulmonary vascular remodeling.

## Discussion

In recent years, there have been clear advances in understanding the pathophysiology of PAH, with more and more therapeutic options available to combat this disease. However, in the absence of an exact cure, the combination of medication to obtain the best possible result is still complex and constantly evolving. Most patients with PAH are of advanced age and, therefore, have different associated comorbidities (8). Thus, interest arises in studying and discovering non-invasive biomarkers to use as a means of screening and diagnosing, to understand the severity and prognosis of the disease, and to monitor the response to therapies (12). Although there are already many known biomarkers, the truth is that an ideal one has not yet been found. An ideal biomarker must be fast, inexpensive, non-invasive, easy to measure, reproducible, and should apply to all classes of disease. Furthermore, it should not have confounding factors, that is, it should not vary with factors such as comorbidities, age, gender, race, etc. Thus, it is difficult to find an ideal biomarker, however, it is possible that a specific set of biomarkers can respond and provide all relevant information about the patient/disease.

The biomarkers addressed in this review article are non-invasive and easy to detect and therefore are of low cost and easy access. All biomarkers investigated have been found to be significantly altered in PAH patients of various subtypes when compared with healthy controls (Supplementary Table 1)—NO and VEGFR-2 appear lower in patient blood when compared to healthy controls, while all other markers are elevated. Moreover, almost all were found to be good indicators of disease severity and/or prognosis (excluding homocysteine, serotonin, MCP-1, oxidized lipids and CD40/CD49L). However, six biomarkers stand out as more informative and potentially more useful, as they were able to show similar predictive value to tools that are currently used in clinical practice, or, in some cases, were able to outperform these tools—these were RDW, osteopontin, angiopoietin-2, endoglin, endostatin and GDF-15 (findings listed in Table 2). Therefore, we consider these specific biomarkers to be the current best candidates for introducing into clinical practice in the near future.

**Table 2:** Major findings regarding novel biomarker performance against validated clinical tools.

**Findings**	**Biomarkers**
Incremental predictive value to REVEAL risk scale	Endostatin (221)
Incremental predictive value to ESC/ERS criteria	Endostatin (221)
Incremental predictive value to NT-proBNP	Red cell distribution width (68), Osteopontin (148), Endoglin (222), Growth differentiation factor-15 (234)
Outperform NT-proBNP	Osteopontin (148), Angiopoietin-2 (180)

However, when interpreting new scientifically evidence, one must be cautious. Most evidence is still new and limited due to the difficulty in forming large patient cohorts, which limits the statistical power of new findings. Moreover, as previously mentioned, PAH pathophysiology is complex, and the role of each biomarker in disease development is yet to be determined. Finally, there is a clear lack of comparative evidence between the novel markers and measures that have been previously validated for patient evaluation, therefore these markers cannot be safely recommended alongside or in replacement of those validated tools; and we were not able to find any research work regarding a multibiomarker approach.

## Conclusion

Although no biomarker has yet been found to be ideal, there are undoubtedly some that stand out for being more informative and that could be part of the set of standard biomarkers to be studied in patients with PAH. Research into PAH biomarkers is an emerging and highly promising scientific field with the potential to revolutionize PAH patient medical care.

## Author Contributions

CB-S, JS-G, and IG: conceptualization. JS-G and IG: writing—original draft preparation and visualization. JS-G, IG, RA, FP, and CB-S: writing—review and editing. CB-S and RA: supervision. All authors have read and agreed to the published version of the manuscript.

## Funding

This research was supported by the Portuguese Foundation for Science and Technology (FCT), under the auspices of the Cardiovascular R&D Center–UnIC [UIDB/00051/2020 and UIDP/00051/2020], projects IMPAcT [PTDC/MED-FSL/31719/2017 and POCI-01-0145-FEDER-031719], NETDIA MOND [POCI-01-0145-FEDER-016385], and DOCnet [NORTE-01-0145-FEDER-000003, NORTE_2020, and under PORTUGAL_2020 Partnership]. JS-G was supported by FCT (UI/BD/150658/2020). ANR-18-CE14-0023 supports HLR.

## Conflict of Interest

The authors declare that the research was conducted in the absence of any commercial or financial relationships that could be construed as a potential conflict of interest.

## Publisher's Note

All claims expressed in this article are solely those of the authors and do not necessarily represent those of their affiliated organizations, or those of the publisher, the editors and the reviewers. Any product that may be evaluated in this article, or claim that may be made by its manufacturer, is not guaranteed or endorsed by the publisher.

## Abbreviations

15-F2t-IsoP: 15-F2-isoprostanes
5-HT: serotonin
5-HTT: serotonin transporter
6MWD: 6-minute walk distance
aa: amino acids
ADM: adrenomedullin
ADMA: asymmetric dimethylarginine
Ang-1: angiopoietin-1
Ang-2: angiopoietin-2
ANP: atrial natriuretic peptide
AVP: arginine vasopressin
BMP9: bone morphogenic protein 9
BNP: brain natriuretic peptide
CD40L: CD40 ligand
cGMP: cyclic guanosine monophosphate
CHD: congenital heart disease
COPD: chronic obstructive pulmonary disease
CRP: C-reactive protein
CTD: connective tissue diseases
CTEPH: chronic thromboembolic pulmonary hypertension
cTn: cardiac troponins
cTnI: cardiac troponin I
cTnT: cardiac troponin T
CysC: cystatin C
ECs: endothelial cells
Eng: endoglin
eNOS: endothelial nitric oxide synthase
ERAs: endothelin receptor antagonists
Es: endostatin
ET: Endothelins
ET-1: endothelin-1
ETA: endothelin type A receptor
ETB: endothelin type B receptor
F2-isoP: F2-isoprostanes
Gal-3: galectin 3
GDF-15: growth differentiation factor-15
HETEs: hydroxyeicosatetraenoic acids
HF: heart failure
HODEs: hydroxyoctadecadienoic acids
hsTnT: high-sensitive cTnT
IDO, indoleamine 2: 3-dioxygenase
IL: interleukin
IL-1R1: interleukin-1 receptor-1
IPAH: idiopathic PAH
MCP-1: monocyte chemoattracting protein-1
mPAP: mean pulmonary artery pressure
MPs: microparticles
mRAP: mean right atrial pressure
MyD88: molecular adapter 88
NF-κB: nuclear factor kappa B
NFAT: nuclear factor of activated T cells
NYHA: New York Heart Association
NO: nitric oxide
NOS: nitric oxide synthase
NT-proBNP: N-terminal prohormone
OPN: osteopontin
PAH: pulmonary arterial hypertension
PAP: pulmonary arterial pressure
PASMCs: pulmonary arterial smooth muscle cells
PDE: phosphodiesterase
PH: pulmonary hypertension
PIM-1: Moloney Murine Leukemia Provirus Integration Site
PVR: pulmonary vascular resistance
PoPH: portopulmonary hypertension
RAP: right atrial pressure
RDW: red blood cell distribution width
RV: right ventricle
sCD40L: soluble CD40 ligand
sEng: soluble endoglin
SMCs: smooth muscle cells
SSc: systemic sclerosis
STAT3: signal transducers and activators of transcription-3
sVEGFR-1: soluble vascular endothelial growth factor receptor type 1
SvO2: mixed venous saturation of oxygen
TMs: tryptophan metabolites
TNF- α: tumor necrosis factor- α
UA: uric acid
VEGF: vascular endothelial growth factor
VEGFR-1: vascular endothelial growth factor receptor type 1
VEGFR-2: vascular endothelial growth factor receptor type 2
VEGF-A: vascular endothelial growth factor A
VEGF-B: vascular endothelial growth factor B
VEGFR: vascular endothelial growth factor receptors
VSMCs: vascular smooth muscle cells
vWF: von Willebrand factor
WHO: World Health Organization.
