# Supplementary material for: An Overview of Circulating Pulmonary Arterial Hypertension Biomarkers
Source: Front Cardiovasc Med. 2022 Jul 14;9:924873. doi: 10.3389/fcvm.2022.924873 (PMC9333554; doi:10.3389/fcvm.2022.924873)
Supplement: Supplementary file 1 [file Table_1.DOCX]

Supplementary Material

**Supplementary Table 1.** A comprehensive table portraying the biomarker categories included in this review and major findings regarding specific PAH subtypes.

| *Biomarker category* | Biomarkers | | PAH group | Alteration | Major findings |
| --- | --- | --- | --- | --- | --- |
| *Cardiac function* | Natriuretic Peptides | BNP | IPAH (32), CTD-PAH (33), PAH associated with congenital systemic-pulmonary shunts (34) | ↑ | Correlation with higher NYHA functional class, lower 6MWD and worse hemodynamic parameters (3,29,32,37) - higher mPAP and lower cardiac output (37). |
|  |  | NT-proBNP | SSc-PAH (39) | ↑ | Correlation with worse PVR, cardiac index, mRAP and mPAP (3). Prediction of patient survival (3,23). |
|  |  |  | IPAH (28) | ↑ |  |
|  |  | ANP | IPAH (40) | ↑ | Correlation with worse mPAP and cardiac output (38). |
|  | Troponin | cTnT | Group I PAH (44,45) | ↑ | Correlation with disease severity and worse survival (45), systolic RV dysfunction and 6MWD (46). |
|  |  | cTnI | Group I PAH (47) | ↑ | Correlation with more severe cardiac hemodynamic and structural abnormalities (47). |
|  | Cystatin C | | Group I PAH (52) | ↑ | Correlation with RV function (higher RV systolic pressure, RV end-diastolic volume and RV end-systolic volume, and lower RV ejection fraction) (52). |
| *Hematopoiesis* | Homocysteine | | Group I PAH (53), CHD-PAH (56) | ↑ | Total plasma levels of homocysteine are increased but no hemodynamic correlation was found (56). |
|  | Red Cell Distribution Width | | Group I PAH (68) | ↑ | Correlation with worse WHO functional class, 6MWD (68) and survival rate (65-68). |
| *Endothelial dysfunction or/and vascular remodeling and damage* | Endothelin-1 | | Group I PAH (72) | ↑ | Correlation with worse RAP, pulmonary artery oxygen saturation (5,74), PVR, and 6MWD (75). |
|  | Adrenomedullin | | Group I PAH (83,85,86) | ↑ | Correlation with disease severity (83,85) and worse mRAP and 6MWD (86). Correlation with NT-proBNP levels and with ESC/ERS and REVEAL risk scores for patient survival (86). |
|  | Copeptin | | Group I PAH (88) | ↑ | Correlation with worse NYHA class and 6MWD (88). Prediction of death, transplantation, and hospitalization (90). Indicator of treatment response (88). |
|  | Nitric Oxide | eNO | IPAH (94-96) | ↓ | Positive correlation with treatment response to Bosentan (94), Epoprostenol (96,98), and Zaprinast (101) |
|  |  | NOx | IPAH (95) | ↓ |  |
|  | Asymmetric dimethylarginine | | IPAH (11,105) | ↑ | Correlation with worse mPAP, PVR index, SvO2, RAP, cardiac index, and survival rate (11,105). |
|  |  |  | CHD-PAH (56,106,107) | ↑ | Correlation with worse RAP, SvO2, cardiac output, cardiac index, and survival rate (56). |
|  | Cyclic Guanosine Monophosphate | Plasma cGMP | Group I PAH (111) | ↑ | Correlation between baseline plasma cGMP disease severity (111). Correlation with treatment response to PDE-5 inhibitors (114-117). |
|  |  | Urinary cGMP | Group I PAH (30,113) | ↑ | Correlation with worse hemodynamic status (113). |
|  | D-Dimers | | IPAH (120-122) | ↑ | Correlation with worse NYHA functional class, mPAP, values of oxygen saturation, 6MWD and 1-year survival (121,122). |
|  |  |  | SSc-PAH (123,125) | ↑ | No diagnostic (124) or prognostic (125) uses have been found. |
|  | Serotonin | | IPAH (134,135) | ↑ | No correlation with disease severity was found (135-137). |
|  |  |  | PAH associated with ventricular septal defect (138) | ↑ |  |
|  | Osteopontin | | Group I PAH (146,147) | ↑ | Correlation with all-cause mortality (146,147), worse mRAP, 6MWD and NYHA functional class (146). Correlation with RV remodeling and dysfunction (148). |
|  |  |  | IPAH (148) | ↑ | Correlation with NT-proBNP at baseline and during follow-up, providing independent and incremental prognostic information (148). |
|  |  |  | SSc-PAH (140), CHD-PAH (149) | ↑ |  |
|  | vonWillebrand Factor | vWF | Group I PAH (120,151-153) | ↑ | Correlation of baseline vWF with worse short- and long-term survival (151,155,158), functional class, 6MWD (159). Correlation with treatment response to prostacyclins and resulting hemodynamic improvements (152,154). Correlation of lower vWF activity with higher risk of death and lung transplant (159). |
|  |  |  | PAH associated with congenital systemic-pulmonary shunts (165) | ↑ | Correlation with higher NT-proBNP (165). |
|  |  | vWF:Ag | CHD-PAH (123,160,161) | ↑ | Correlation with higher mortality (160,161). |
|  |  |  | Group I PAH (154-157) | ↑ |  |
|  | Microparticles | | Group I PAH (174,175,177) | ↑ | Subtypes: EMPs (CD105^+^, tissue factor^+^ (169), PECAM^+^, VE-cadherin^+^, E-Selectin^+^ (170)), LMPs (170) and urinary MPs (172). Correlation of tissue factor^+^ with worse disease severity (measured by 6MWD and NYHA class of 3 or higher) (169). Correlation of PECAM^+^ and VE-cadherin^+^ with worse hemodynamic severity (105). Correlation of E-Selectin+ with higher negative outcomes (171). Correlation of urinary MPs with worse RV function (measured by tricuspid annular plane systolic excursion) (172). |
|  |  |  | IPAH (173-175) | ↑ | Subtypes: LMPs derived from T-cells (173), PMPs (CD42a and CD42b^+^) (174), EMPs (CD31^+^/CD42b^-^) (175). No correlation between EMPs (CD31^+^/CD42b) and clinical improvement was found (175). |
|  |  |  | SSc-PAH (175,176) | ↑ | Subtypes: EMPs (CD31^+^/CD42b^-^) (180) and MPs VE-cadherin^+^ (181). VE-cadherin^+^ for the prediction of PAH development (181). Correlation between EMPs (CD31^+^/CD42b^-^) and clinical improvement was found (180). |
|  |  |  | Eisenmenger Syndrome (177) | ↑ | Subtypes: MPs VE-cadherin^+^ and CD146^+^ (177). |
| *Angiogenesis* | Angiopoietins | Ang-2 | IPAH (180,181) | ↑ | Correlation with worse mRAP, PVR, NYHA functional class, cardiac index and SvO2. Prediction of mortality (180) and indicator of treatment response (180,181). |
|  |  |  | SSc-PAH (182) | ↑ |  |
|  |  | Ang-1 | IPAH (180,183) | ↑ |  |
|  | Vascular endothelial growth factor | sVEGFR-1 | Group I PAH (188-190) | ↑ | Correlation with worse functional class and disease severity (188,190), higher patient survival (188), and adverse events (191). |
|  |  |  | IPAH (189) | ↑ | Correlation with treatment response (189). |
|  |  |  | SSc-PAH (189,192) | ↑ | Prediction of treatment response (189) and PAH development (189,192). Correlation with worse RV systolic pressure and capacity of diffusing carbon monoxide (192). |
|  |  | VEGF | CHD-PAH (106) | ↑ | VEGF expression, not VEGF ligand serum levels, is elevated and predicts worse outcomes post-surgical treatment (106). |
|  |  |  | IPAH (180,187,189,193) | ↑ |  |
|  |  |  | SSc-PAH (162,189,194) | ↑ |  |
|  |  | VEGFR-2 | Group I PAH (187) | ↓ |  |
|  |  | VEGFR | IPAH (184,185) | ↑ |  |
|  | Endoglin | | Group I PAH (188) | ↑ | Higher sensitivity for the presence of PAH and prediction of NYAH functional class than NT-proBNP. Correlation with worse patient survival (188). |
|  |  |  | SSc-PAH (196) | ↑ |  |
|  | Bone morphogenic protein 9 | | Hereditary PAH (206-209) | ↓ |  |
|  |  |  | IPAH (210-212) | ↓ |  |
|  |  |  | PoPH (214-216) | ↓ | Prediction of with worse transplant-free survival. Distinguished PoPH from liver disease without PAH. |
|  | Endostatin | | IPAH (220,221) | ↑ | Correlation with hemodynamic parameters (higher mPAP and PVR, lower cardiac index and cardiac output) worse functional class and reduced exercise tolerance (220,223). Could predict disease mortality (223). Improved predictive performance of REVEAL, ESC/ERS, (221) and NT-proBNP (222). |
|  |  |  | CHD-PAH (222) | ↑ | Correlation with worse hemodynamics and functional capacity, and with several echocardiographic alterations predicting RV dysfunction (222). Improved predictive performance of REVEAL, ESC/ERS, (221) and NT-proBNP (222). |
|  |  |  | CTD-PAH (221) | ↑ | Correlation with worse disease severity (measured by mRAP, mPAP, PVR, 6MWD, pulmonary artery compliance and stroke volume) and mortality (221). In SSc, appeared to predict the development of PAH (224). Improved predictive performance of REVEAL, ESC/ERS, (221) and NT-proBNP (222). |
| *Inflammation/oxidative stress* | C-reactive protein | | Group I PAH (227,228) | ↑ | Correlation with worse NYHA functional class, 6MWD, RAP, and survival. Prediction of outcome and response to therapy (228). |
|  | Growth differentiation factor-15 | | IPAH (234) | ↑ | Correlation with more severe disease and poor prognosis (234). Correlation with a baseline NT-proBNP levels (234). GDF-15 levels in combination with NT-proBNP improved the detection of high-risk cases (234). |
|  | Uric acid | | Group I PAH (240,243) | ↑ | Correlation with worse NYHA functional class (240,243), total pulmonary resistance (240), mortality (240,243), cardiac output (240) and 6MWD (243). |
|  |  |  | IPAH (244) | ↑ |  |
|  | Monocyte chemoattracting protein-1 | | IPAH (248,249) | ↑ | No correlation with hemodynamic parameters was found (247,249). Indicator of response to treatment with Epoprostenol (249). |
|  |  |  | CTD-PAH (247) | ↑ |  |
|  | Galectin-3 | | Group I PAH (256,258,259) | ↑ | No correlation with structural and functional parameters of RV. Prediction of mortality (256,258). Increases linearly in the five risk levels on the REVEAL 2.0 risk scale (259). |
|  | Interleukins | | Group I PAH (220,229,230) | ↑ | Interleukines:IL-1β and IL-6 – (252,262) IL-2, IL-4, IL-8, IL-10 and IL-12p70 (261). No correlation with hemodynamic parameters was found (252,261). Prediction of worse patient survival (261). |
|  |  |  | Pediatric PAH (191) | ↑ | Correlation with patient outcome (191). |
|  |  |  | IPAH (265,263) | ↑ | Interleukines: IL-21 (265), IL-1R1 and MyD88 (263). |
|  | Isoprostanes | Plasma 15-F2t-IsoP | IPAH (272) | ↑ | Correlation with worse WHO functional class, 6MWD, SvO2, mRAP, and BNP levels (272). |
|  |  | Urinary F2-isoP | Group I PAH (268,271,272,274) | ↑ | Correlation with lower pulmonary vasoreactivity (274) and increased hazard of death (268). |
|  | Oxidized lipids | | Group I PAH (277-279) | ↑ | Subtypes: Oxidized fatty acids 5-, 12-, and 15-HETE, and 9- and 13-HODE (277-279). |
|  | CD40/CD49L | sCD40L | Group I PAH (289) | ↑ | Turning off the CD40 pathway may have advantages in therapies for PAH, namely endothelial progenitor cell transplantation (288). |
| *Metabolic* | Tryptophan Metabolites | IDO-TMs | Group I PAH (291, 293-295) | ↑ | Correlation with worse right ventricle-pulmonary vasculature dysfunction (measured by resting RAP, PVR, and change in cardiac output during exercise) (291). Correlation with worse exercise PVR and PAP (293) and with negative patient outcomes (294). |
|  | Ghrelin | Total plasma ghrelin | IPAH (299) | ↑ | Correlation with worse right ventricular hemodynamics (measured by RV diameter and pulmonary artery systolic pressure). Correlation with N-BNP, ET-1 and NO measures (299). |
|  |  | Acyl-ghrelin | CHD-PAH (300,301) | ↑ | Correlation with worse pulmonary artery diastolic pressure, RV systolic pressure, mPAP, and pulmonary artery trunk diameter. Correlation with ET-1 and NO measures (300). |
| *Transcriptional regulators and oncogenes expression* | PIM-1 | | Group I PAH (306) | ↑ | Correlation of PIM-1 expression with higher pulmonary artery remodeling and pressure. Correlation with worse disease severity (306). |
|  |  |  | CTD-PAH, IPAH (309) | ↑ | Correlation with disease severity and prediction of mortality (309). |
